# Comparative Mitogenomics and Phylogeny of Geotrupidae (Insecta: Coleoptera): Insights from Two New Mitogenomes of Qinghai–Tibetan Plateau Dung Beetles

**DOI:** 10.3390/biology15020164

**Published:** 2026-01-16

**Authors:** Huan Wang, Sha-Man Ai, Han-Hui-Ying Lv, Shi-Jun Li, Yu-Xiang Wang, Ming-Long Yuan

**Affiliations:** 1 College of Grassland Science, Xinjiang Agricultural University, Urumqi 830052, China; 13993665785@163.com; 2 State Key Laboratory of Herbage Improvement and Grassland Agro-Ecosystems, College of Pastoral Agricultural Science and Technology, Lanzhou University, Lanzhou 730020, China; aishm2023@lzu.edu.cn (S.-M.A.); lvhhy2025@lzu.edu.cn (H.-H.-Y.L.); 18143763078@163.com (S.-J.L.)

**Keywords:** dung beetles, Qinghai–Tibetan Plateau, mitochondrial genomes, phylogeny, non-coding regions

## Abstract

The dung beetle family Geotrupidae (Scarabaeoidea) plays a vital ecological role in nutrient cycling and soil health, yet the scarcity of complete mitochondrial genome (mitogenome) data has hindered phylogenetic and comparative studies within this family. Here, we sequenced, assembled, and annotated the first complete mitogenomes of *Geotrupes stercorarius* and *Phelotrupes auratus*, collected from the Qinghai–Tibetan Plateau. Comparative analysis of these two novel mitogenomes with eight existing mitogenomes revealed conserved architectural features across Geotrupidae, such as gene arrangement, tRNA secondary structures, and small intergenic spacers. Nucleotide composition was largely conserved, though marked divergence occurred at the third codon positions. Substantial structural variation was observed in non-coding regions, particularly in the control region and the *nad2*-*trnW* spacer. Evolutionary analyses indicated strong purifying selection across all protein-coding genes, with no evidence of widespread positive selection linked to high-altitude adaptation. Phylogenetic reconstruction consistently recovered the relationships (Bolboceratinae, (Lethrinae, Geotrupinae)), with *Anoplotrupes* and *Geotrupes* forming sister genera within Geotrupinae. This study provides additional mitogenomic resources and a well-supported phylogenetic framework for Geotrupidae, resolving key taxonomic uncertainties and establishing a basis for future evolutionary and ecological research.

## Simple Summary

The dung beetle family Geotrupidae (Scarabaeoidea) is crucial for nutrient cycling and soil health, but limited complete mitochondrial genome (mitogenome) data has slowed its phylogenetic and comparative studies. In this work, we obtained the first complete mitogenomes of two high-altitude Geotrupidae species (*Geotrupes stercorarius* and *Phelotrupes auratus*) from the Qinghai–Tibetan Plateau. By comparing these new mitogenomes with eight existing ones, we found conserved genomic features (e.g., gene arrangement) in Geotrupidae, while variations appeared in non-coding regions. Evolutionary analyses indicated strong purifying selection on protein-coding genes, with no widespread positive selection linked to high-altitude adaptation. Phylogenetic reconstruction consistently recovered the relationships (Bolboceratinae, (Lethrinae, Geotrupinae)). This study provides precious mitogenomic resources and a clear phylogenetic framework for Geotrupidae.

## 1. Introduction

The Geotrupidae (dung beetles) is a family within the superfamily Scarabaeoidea (Coleoptera: Polyphaga), comprising approximately 1000 known species with a global distribution across temperate and tropical regions. Both adults and larvae of Geotrupidae are predominantly coprophagous or saprophagous [1,2]. By feeding on, transporting, and burying feces and decaying organic matter, they play an irreplaceable role in nutrient cycling, soil aeration, and ecosystem functioning, making them ecologically vital insects [2,3].

Despite their ecological importance, the taxonomy and phylogeny of Geotrupidae remain controversial. Traditional morphology-based classifications recognized three subfamilies, i.e., Geotrupinae, Lethrinae, and Bolboceratinae [1]. However, the taxonomic status of some groups, particularly Bolboceratinae, is unstable, with proposals to elevate it to family rank [4,5], while larval morphology and molecular data often support its taxonomic rank as a subfamily within Geotrupidae [1,6]. These inconsistencies highlight the limitations of morphological characters, which may be prone to convergence, and of early molecular studies that relied on limited genetic loci with insufficient phylogenetic signal to confidently resolve higher-level relationships [7].

The mitochondrial genome (mitogenome), characterized by structural conservation, a moderate evolutionary rate, and maternal inheritance, has become a cornerstone molecular marker for insect phylogenetics and taxonomy [8]. Within the Scarabaeoidea, mitogenomic data have provided critical insights into gene order evolution, selective pressures, and subfamily-level systematics for families such as Scarabaeidae. In contrast, mitogenomic resources for Geotrupidae are notably scarce. Most available data consist of partial gene sequences, and a comprehensive comparative analysis based on complete mitogenomes—essential for clarifying deep phylogenetic nodes—is still lacking [9,10].

*Geotrupes stercorarius* (Linnaeus, 1758) and *Phelotrupes auratus* (Motschulsky, 1857) (the tribe Geotrupini) are widespread species exhibiting notable morphological and ecological variation, making them valuable models for studying diversification within the Geotrupidae family [11]. Notably, populations of these species from the Qinghai–Tibetan Plateau (QTP) represent unique lineages inhabiting high-altitude environments. Previous molecular studies on these taxa have been restricted to fragments of *cox1* or ribosomal DNA [10,12,13,14], which cannot reveal whole mitogenome architecture—including gene order and control region structure—nor provide robust phylogenetic resolution. Although some studies have explored phylogenetic relationships using chemical or morphological characteristics [14,15,16], the evolutionary relationship between the genera *Phelotrupes* and *Geotrupes* remains unclear [2,10,14,17].

Here, we sequenced and annotated the complete mitogenomes of *Geotrupes stercorarius* and *Phelotrupes auratus* for the first time, with samples originating from the QTP. By combining these with eight sequenced Geotrupidae mitogenomes from GenBank, we conducted a comprehensive comparative mitogenomic analysis across the three subfamilies (Geotrupinae, Bolboceratinae, and Lethrinae). Our objectives were to: (1) characterize the structural features, nucleotide composition, and non-coding regions of these mitogenomes; (2) reconstruct a robust phylogeny of Geotrupidae using multiple datasets and inference methods; and (3) clarify the systematic positions of *Geotrupes* and *Phelotrupes* within Geotrupinae. This study provides essential genomic resources and a refined phylogenetic framework for future investigations into the evolution and adaptation of Geotrupidae.

## 2. Materials and Methods

### 2.1. Sampling and DNA Extraction

Adult specimens of *Geotrupes stercorarius* and *Phelotrupes auratus* were collected from alpine meadows in Yushu County (~4000 m) and Menyuan County (~3000 m), Qinghai Province, China, during July and August 2023, respectively (Table S1). Specimens were initially preserved in absolute ethanol in the field and subsequently stored at −80 °C at Lanzhou University. Total genomic DNA was extracted from the thoracic muscle tissue of individual specimens using the DNeasy Blood & Tissue Kit (Qiagen, Hilden, Germany). DNA concentration and purity were assessed using a NanoDrop ND-1000 spectrophotometer (Thermo Fisher Scientific, Waltham, MA, USA), and integrity was verified by 1.2% agarose gel electrophoresis.

### 2.2. Mitogenome Sequencing, Assembly, and Annotation

Paired-end sequencing libraries (2 × 150 bp) were constructed and sequenced on an Illumina NovaSeq 6000 platform by Wuhan Bena Technology Co., Ltd. (Wuhan, China). Raw reads were subjected to quality control using fastp v0.23.2 [18] to remove adapters and low-quality bases. High-quality reads were de novo assembled using GetOrganelle v1.7.1 [19]. Candidate mitochondrial contigs were identified by BLASTn (BLAST+ v2.15.0, National Center for Biotechnology Information, Bethesda, MD, USA) searches against the NCBI nucleotide (nt) database.

The complete circular mitogenomes were annotated using the MitoZ v3.6 [20] with the invertebrate genetic code. The boundaries of protein-coding genes (PCGs) were manually verified by identifying open reading frames and comparing them with homologous sequences from related dung beetles. The secondary structures of transfer RNA (tRNA) genes were predicted by MitoZ v3.6 [20] and manually adjusted to conform to the typical cloverleaf structure. The two ribosomal RNA (*rrnL* and *rrnS*) genes were annotated based on sequence homology. Circular genome maps were generated using CGView [21].

### 2.3. Comparative Mitogenomic Analysis

Comparative analyses were performed on the ten Geotrupidae mitogenomes. In addition to the newly sequenced mitogenomes, the mitogenome sequences of eight other Geotrupidae species were downloaded from the National Center for Biotechnology Information (NCBI) GenBank database (Table S2). Nucleotide composition (A, T, G, C content), AT-skew [(A−T)/(A+T)], and GC-skew [(G−C)/(G+C)] [22] were calculated for the whole genome, individual genes, and codon positions using MEGA v12.1.0 [23]. Relative synonymous codon usage (RSCU) for the 13 PCGs was calculated with MEGA v12.1.0 [23]. The effective number of codons (ENC) and codon bias index (CBI) were computed using DnaSP v6.12.3 [24], and their correlations with GC content and GC content at the third codon position (GC3%) were analyzed.

To assess interspecific genetic variation, the nucleotide diversity (*Pi*) of each PCG across all species was calculated using DnaSP v6.12.3 [24] with a sliding window approach (window length: 100 bp; step size: 25 bp). The rates of non-synonymous (*Ka*) and synonymous (*Ks*) substitutions for each PCG were estimated using MEGA v12.1.0 [23]. The codeml program in PAML v4.10.9 was used to analyze the selective pressures for each PCG under type of branch-site models using a maximum-likelihood approach [25]. Two Geotrupidae species (*G. stercorarius* and *P. auratus*) inhabiting the QTP were used as the foreground branch. Positively selected sites were detected when *ω* > 1 and the LRT was significant (*p* < 0.05) [25]. The Bayes empirical Bayes (BEB) method was used to calculate posterior probabilities for site classes to determine which codon positions have experienced positive selection (*ω* > 1) [26].

Tandem repeats in the control region and other non-coding regions were identified using the Tandem Repeats Finder program [27] with default parameters. Schematic diagrams of repeat structures were generated for visual comparison.

### 2.4. Phylogenetic Analysis

Three datasets were constructed for phylogenetic inference: (1) P123: concatenated nucleotide sequences of all 13 PCGs (11,130 bp); (2) P123AA: concatenated amino acid sequences of the 13 PCGs (3710 amino acids); (3) P123R: concatenated P123 nucleotides plus the two rRNA genes (*rrnL* and *rrnS*) (13,259 bp). Each PCG was aligned separately at the codon level using MAFFT v7.505 [28], while rRNA genes were aligned using the Q-INS-i algorithm in MAFFT, which considers secondary structure. Poorly aligned positions were removed using Gblocks v0.91b [29] with relaxed parameters. *Dynastes satanas* (OQ998898) and *Xylotrupes sumatrensis* (OK484316) were used as outgroups (Table S2).

Substitution saturation tests performed using DAMBE v7.0.35 [30] indicated no significant saturation in the 13 PCGs (Table S3). The best-fit partitioning schemes and corresponding substitution models for each dataset were selected using the -m MFP+MERGE function in IQ-TREE v2.2.0 [31] via the ModelFinder option [32], which minimizes the Bayesian Information Criterion (BIC). The best schemes and evolutionary models are provided in Table S4.

Maximum Likelihood (ML) phylogenies were reconstructed using RAxML v8.2.12 [33] with 10,000 ultrafast bootstrap replicates [34]. Bayesian Inference (BI) was performed using MrBayes v3.2.7 [35] on the CIPRES Science Gateway. For BI, two independent runs of four Markov Chain Monte Carlo (MCMC) chains each were conducted for 10 million generations, sampling every 1000 generations. The first 25% of samples were discarded as burn-in after confirming convergence (average standard deviation of split frequencies < 0.01). Phylogenetic trees were visualized and annotated using TreeView v2.2.0 [36].

## 3. Results

### 3.1. General Features and Comparative Analysis of Geotrupidae Mitogenomes

**Figure 1:**
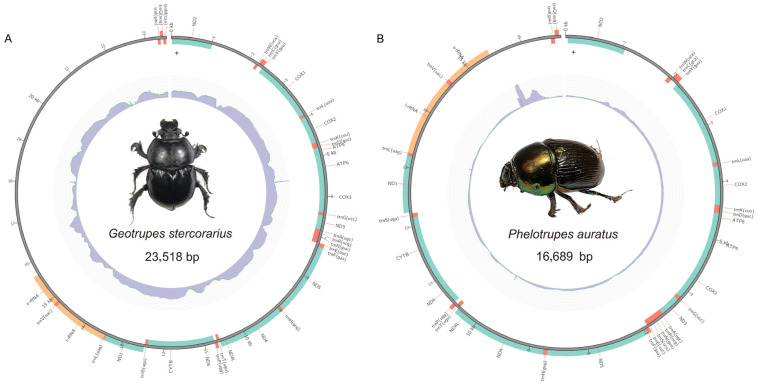
Circular maps of the mitogenomes of *Geotrupes stercorarius* (**A**) and *Phelotrupes auratus* (**B**). The maps depict the organization of the 37 mitochondrial genes: 13 protein-coding genes (PCGs, green), 22 transfer RNA genes (tRNAs, orange), and two ribosomal RNA genes (rRNAs, yellow). The major non-coding control region is shown in gray. Genes on the outer strand are transcribed clockwise.

We sequenced and assembled the complete mitochondrial genomes of *Geotrupes stercorarius* (23,518 bp) and *Phelotrupes auratus* (16,689 bp) from the QTP (Figure 1). Combined with eight published mitogenomes retrieved from GenBank, our comparative analysis encompassed ten species across the three subfamilies of Geotrupidae (Geotrupinae, Bolboceratinae, and Lethrinae).

**Figure 2:**
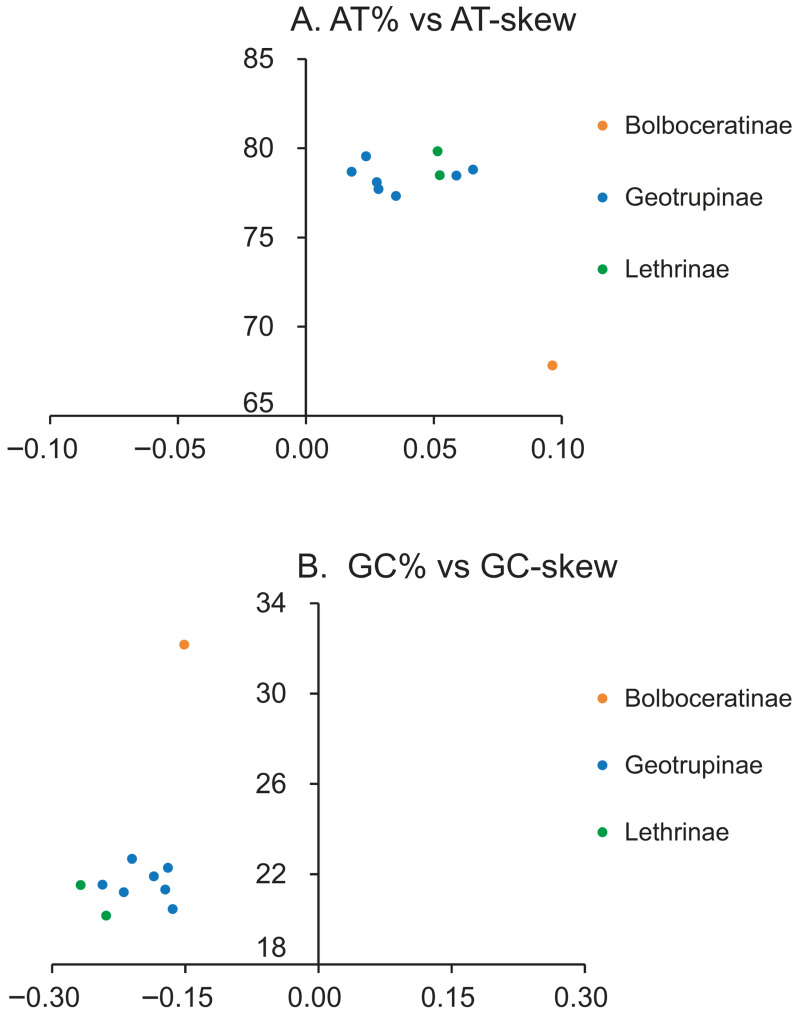
Nucleotide composition skews in Geotrupidae mitogenomes. (**A**) AT-skew plotted against overall AT content. (**B**) GC-skew plotted against overall GC content. Values were calculated for the entire major strand (J-strand) of each complete mitochondrial genome.

All Geotrupidae mitogenomes contained the typical 37 genes (13 PCGs, 2 rRNAs, 22 tRNAs) and a control region, with no gene rearrangements detected (Table S5). Genome lengths varied considerably, ranging from 12,501 bp in *Anoplotrupes stercorosus* (MT862428) to 24,944 bp in *Lethrus scoparius*, primarily due to length polymorphism in the control region (Figure S1). All mitogenomes exhibited a strong AT bias, with total A+T content ranging from 67.83% in *Bolboceratex* sp. to 79.83% in *Lethrus scoparius*. The subfamily Bolboceratinae showed significantly lower total A+T content than Geotrupinae and Lethrinae. AT-skew was consistently positive, while GC-skew was negative across all species (Figure 2).

### 3.2. Nucleotide Composition, Codon Usage, and Evolutionary Rates

In protein-coding genes (PCGs), the A+T content was highest at the third codon position (94.18% on average), significantly exceeding that at the first and second positions (Figure S2). Notably, the two newly sequenced plateau species did not exhibit extreme A+T content compared to their congeners, and no significant differences in A+T content were observed across the three subfamilies.

Nucleotide diversity of 13 PCGs differed significantly across genera and individual genes. The genus *Lethrus* displayed markedly higher *Pi* values than the other three genera for most genes (e.g., *atp6*, *nad1*, *nad3*) (*p* < 0.001), with *nad3* reaching the peak (Figure S3A). Conversely, *Pi* values for *Anoplotrupes* were generally close to zero (Figure S3). The two newly sequenced species showed lower nucleotide diversity than the other analyzed species; among their PCGs, *atp8* had the highest polymorphism (*Pi* = 0.173), while *nad1* exhibited the lowest (*Pi* = 0.107). In contrast, among the other species, *nad6* displayed the highest diversity (*Pi* = 0.251), whereas *cox1* had the lowest (*Pi* = 0.163) (Figure S4).

The evolutionary rates (*Ka*/*Ks*) of all 13 PCGs were less than 1 (range: 0.05–0.43), indicating they are under strong purifying selection. The *atp8* gene showed the highest evolutionary rate, while *cox1* was the most conserved (Figure S5). A significant negative correlation was observed between *Ka*/*Ks* values and the G+C content of PCGs (*R*^2^ = 0.73, *p* < 0.01; Figure S6). In the branch-site model analysis, when *P. auratus* was designated as the foreground branch, significant signals of positive selection were detected in *atp6* (*p* = 0.002; Table S6). When *G. stercorarius* was set as the foreground branch, only a single codon in the *nad5* gene showed a marginally significant signal (*p* = 0.051; Table S6).

**Figure 3:**
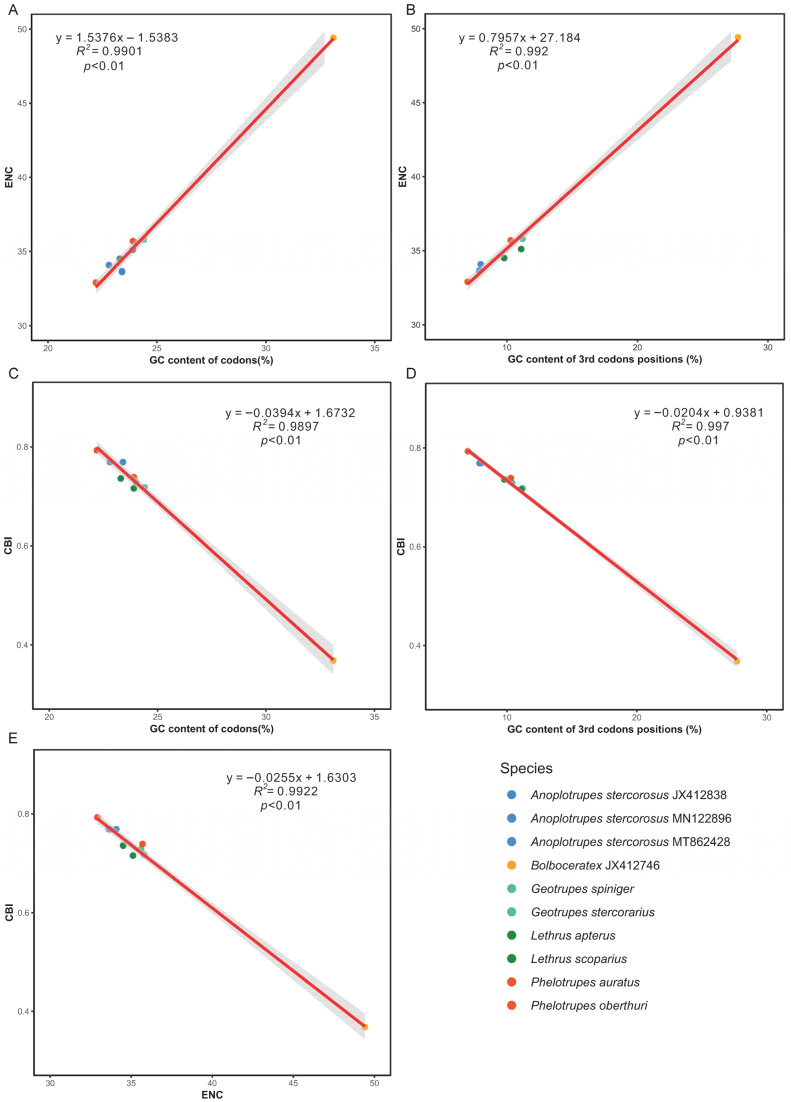
Analysis of codon usage bias in Geotrupidae mitogenomes. Correlations between (**A**) effective number of codons (ENC) and overall GC content (GC%), (**B**) ENC and third-position GC content (GC3%), (**C**) codon bias index (CBI) and GC3%, (**D**) CBI and GC%, and (**E**) ENC and CBI. The red line represents the linear regression fit of the relationship. The shaded area around the line indicates the 95% confidence interval of the regression. Each point represents one species, and the same color denotes one genus.

Codon usage analysis revealed a preference for AT-ending codons (Figure S7; Table S7). The effective number of codons (ENC) showed a strong positive correlation with both overall GC% and GC3%, while the codon bias index (CBI) was negatively correlated with these metrics (Figure 3). All species plotted below the expected ENC curve, indicating that factors beyond mutational bias could influence codon usage (Figure S8).

### 3.3. Structural Conservation of RNA Genes and Non-Coding Regions

**Figure 4:**
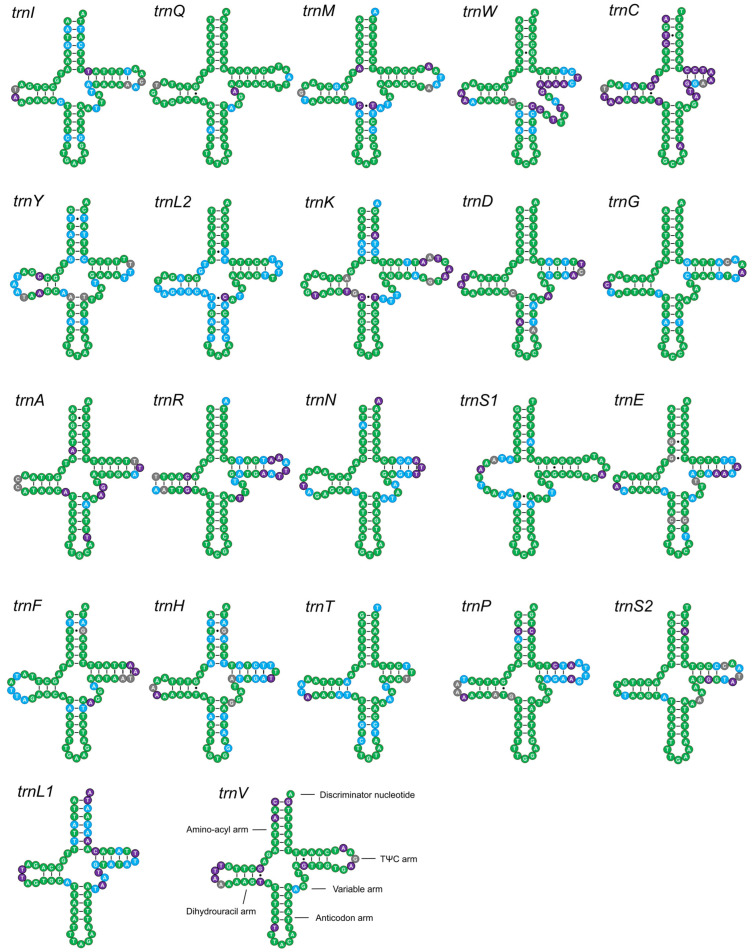
Predicted secondary structures of the 22 mitochondrial tRNA genes of Geotrupidae. tRNAs are displayed in their genomic order, starting with *trnI*. Nucleotide conservation is color-coded: green (conserved across all Geotrupidae), blue (conserved within Geotrupinae), purple (conserved within the genus *Geotrupes*), and gray (not conserved). Watson–Crick pairings are indicated by bars, and G-U wobble pairs by dots.

The secondary structures of the 22 tRNAs were highly conserved within Geotrupidae. All exhibited the typical cloverleaf structure except *trnS1*, which lacked the DHU arm. Sequence conservation rates for tRNAs ranged from 54.41% (family level) to 100% (within some subfamilies) (Figure 4). The lengths of *rrnS* and *rrnL* genes were 587–791 bp and 1266–1310 bp, respectively, with A+T contents above 78%. Sequence conservation was highest within each subfamily, particularly in Lethrinae.

**Figure 5:**
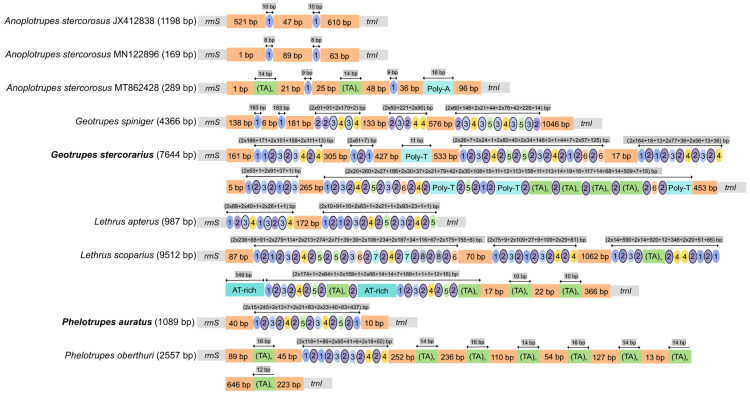
Structural organization of the mitochondrial control region in Geotrupidae. The region located between *rrnS* and *trnI* is depicted for representative species. Key features are annotated: orange blocks (non-repetitive spacers), colored ellipses with numbers (unique tandem repeat units), bordered ellipses (inter-repeat spacers), green blocks (TA motifs), light blue blocks (poly-A/poly-T/AT-rich stretches), and gray blocks (scale). Different numbers and colors indicate different tandem repeat units.

The control region (CR), located between *rrnS* and *trnI*, was the major source of genome size variation, containing AT-rich segments and repetitive sequences. Its length varied dramatically from 169 bp (*Anoplotrupes stercorosus* MN122896) to 9512 bp (*Lethrus scoparius*) (Figure 5). While Bolboceratinae species lacked extensive AT-rich repeats, Lethrinae possessed a pronounced AT-rich profile and exhibited a greater number of repeat units than Geotrupinae (Figure 5). Notably, *Lethrus scoparius* had the longest repeat unit (279 bp), resulting in the longest control region (9512 bp) among all species (Table S8). The two species from the QTP exhibited considerable variation in control region length, with *G. stercorarius* displaying a more complex structure and a greater number of distinct repetitive units than *P. auratus* (Figure 5). Additionally, a non-coding region with structural similarity to the CR (containing repetitive units) was identified between *nad2* and *trnW* in both Geotrupinae and Lethrinae, with the longest one (2538 bp) presented in *Geotrupes spiniger* (Figure S9, Table S9). The repeat copy number in Geotrupinae was higher than that in Lethrinae, and a poly-A structure in this region was unique to the genus *Geotrupes* (Figure S9). Notably, *G. spiniger* harbored the longest repeat unit (139 bp), resulting in the longest between *nad2* and *trnW* spacer (2538 bp) across all taxa (Table S9). A short, highly conserved intergenic spacer was consistently found between *trnS2* and *nad1* across all species, ranging from 7 bp to 23 bp in length (Figure S10).

### 3.4. Mitochondrial Phylogeny of Geotrupidae

**Figure 6:**
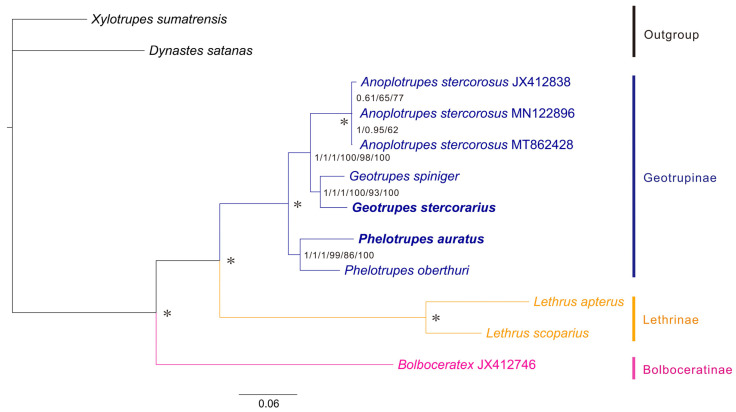
Phylogenetic relationships within Geotrupidae inferred from mitochondrial genomes. Numbers from left to right are Bayesian posterior probabilities (PP) and ML bootstrap (BS) values of each of the three datasets (P123, P123AA and P123R). Asterisk (*) indicates PP = 1.0 and BS = 100.

Phylogenetic analyses based on the three datasets (P123, P123AA, P123R) using both ML and BI methods yielded largely congruent and well-supported topologies (Figure 6). The resulting phylogeny strongly supports the monophyly of each of the three subfamilies (all Bayesian Posterior Probabilities, PP = 1.0). Bolboceratinae was recovered as the earliest-diverging lineage, whereas Lethrinae and Geotrupinae formed a sister clade with strong support (PP = 1.0). Within Geotrupinae, *Geotrupes stercorarius* formed a well-supported clade with *G. spiniger*. This *Geotrupes* clade was recovered as the sister group to the genus *Anoplotrupes* (PP = 1.0), with the genus *Phelotrupes* positioned as the basal lineage within the subfamily (Figure 6).

## 4. Discussion

### 4.1. A Robust Phylogenetic Framework for Geotrupidae and Its Taxonomic Implications

The primary contribution of this study is the establishment of a well-supported mitochondrial phylogenomic framework for Geotrupidae. Our analyses based on multiple datasets and inference methods consistently recovered the monophyly of the two subfamilies (Geotrupinae, Lethrinae) with strong supports (Figure 6), which was consistent with previous studies based on larval morphological characteristics and partial single-gene sequences [17,37]. The taxonomic status of Bolboceratinae remains a matter of debate to this day [38,39]. The recovered topology—(Bolboceratinae, (Lethrinae, Geotrupinae))—was consistent with previous studies [17] and offered an evolutionary narrative congruent with ecological shifts. The basal position of the predominantly saprophagous Bolboceratinae suggested that coprophagy, the defining habit of Geotrupinae and Lethrinae, is a derived trait within the family. This supports the hypothesis of an evolutionary transition from generalist saprophagy to specialized dung-feeding, a key innovation that may have driven diversification in this group [11,40].

Crucially, by incorporating *G. stercorarius* and *P. auratus* into a mitogenomic phylogeny for the first time, we resolved the relationships among *Geotrupes*, *Phelotrupes*, and *Anoplotrupes* within Geotrupinae. This result further supports that these three genera belong to the same evolutionary lineage [37], which is consistent with their shared morphological characteristics, such as coprophagous nesting behavior [6,41,42].

The unstable positioning of conspecific sequences and the generally higher node support in BI trees compared to ML BS values both pointed to limitations in mitochondria data for resolving shallow divergences. This may arise from insufficient accumulation of mitochondrial genetic variation following recent population divergence or mitochondrial gene flow between related populations, which can obscure lineage boundaries [43,44]. These issues highlight the inherent limitations of mitogenomes in resolving very recent divergences or complex population histories [8,45], as indicated by the *Carbula humerigera* complex, where mitogenomes failed to delineate recent species accurately compared to nuclear genes [46]. These sequences likely represent distinct geographical populations where incomplete lineage sorting or mitochondrial introgression obscures phylogenetic signal [47]. This underscores the necessity of future studies to incorporate multi-locus nuclear data to fully resolve species-level relationships and population structure within Geotrupidae.

### 4.2. Conserved and Variable Features in Geotrupidae Mitogenomes

Our comparative analysis reveals a highly conserved mitogenomic architecture across Geotrupidae, consistent with the pattern observed in most insects [10]. No gene rearrangements were detected, and the core set of 37 genes and their order are maintained. The most striking source of genomic variation is the extreme length polymorphism of CR, which accounted for nearly all size differences between species (e.g., ~9.5 kb in *Lethrus scoparius* vs. ~1 kb in others). CR expansion via tandem repeats is common in beetles and these repetitive regions hold potential as population-level markers [48], though their functional and evolutionary significance in Geotrupidae warrants further study. Concurrently, the number of tandem repeat sequences within this segment correlates with the size of CR, a phenomenon also observed in other species [49]. Moreover, a distinct inverse relationship existed between sequence size and the number of tandem repeats [50]. In *G. stercorarius* and *P. auratus*, two repeat sequences were arranged in parallel, clearly demonstrating a sequence pattern that competitively accumulated the maximum possible number of copies [51]. The discovery of an extended, repeat-containing non-coding region between *nad2* and *trnW* in some species (notably *Geotrupes spiniger*) was intriguing. While its function was unknown, analogous regions in other insects have been implicated in replication regulation [12,52], suggesting a target for future functional investigation.

Strong AT bias and codon usage preferences are pervasive, with the third codon position being exceptionally AT-rich. This is attributed to mutational pressure and is a common feature of insect mitogenomes, likely reflecting replication or transcription-associated strand asymmetry [8,12]. Notably, the two newly sequenced species from the QTP did not exhibit markedly divergent base composition or codon usage patterns from their lowland congeners, suggesting that the extreme mitochondrial genomic environment is primarily shaped by deep phylogenetic history rather than recent altitude-associated adaptation [53].

All PCGs are under strong purifying selection (*Ka*/*Ks* << 1), with the expected pattern of *cox1* being the most conserved and *atp8* the most rapidly evolving. The high conservation of *cox1* reaffirms its utility as a DNA barcode for species identification in this family [54].

### 4.3. Insights and Perspectives on High-Altitude Lineages and Future Research

A key objective of this study was to examine the mitogenomes of high-altitude dung beetle lineages for signatures of adaptation. Our PAML analyses did not detect widespread positive selection across the 13 PCGs when considering the two QTP species as a foreground branch, suggesting that high-altitude adaptation in these *Geotrupes* and *Phelotrupes* populations may not be strongly mediated by amino acid changes in mitochondrial proteins [55,56]. The extreme conditions of the QTP (hypoxia, low temperature, strong UV radiation) exert strong selective pressure on energy metabolism, membrane stability, and genetic integrity [57,58]. Adaptive evolution to these stressors may therefore involve multiple synergistic mechanisms beyond positive selection in protein-coding genes, such as: (1) nuclear-regulated changes in mitochondrial function or biochemical adjustments (Comparative studies on other QTP endemics, like mammals and insects, reveal both conserved and lineage-specific adaptive strategies to these extremes [59,60]); (2) structural variations in non-coding regions influencing replication or transcription efficiency; and (3) biochemical or physiological adjustments (e.g., metabolic rate modulation) not requiring fixed changes in PCG sequences [61,62]. The single signal of positive selection on *atp6* in one species (*Phelotrupes auratus*) when analyzed alone requires cautious interpretation and validation with larger population-level sampling, as it could reflect lineage-specific innovation or a false positive [63,64].

Further study is needed by expanding taxa sampling and integrating diverse data (e.g., nuclear genomic, transcriptomic, or phenotypic) to comprehensively test adaptation hypotheses, resolve shallow nodes, and understand the full evolutionary history of Geotrupidae. Despite these limitations, our work provides valuable mitochondrial genome resources—the first complete mitogenomes for two QTP dung beetle species and a robust subfamily-level phylogeny—that will facilitate and guide these future investigations.

## 5. Conclusions

This study provides the first complete mitogenomes for Geotrupidae from the QTP. Our comparative analyses confirmed a conserved gene order and composition across the family, with the control region identified as the primary source of structural variation. The mitogenomic phylogeny robustly resolved subfamily relationships, supporting a phylogeny of (Bolboceratinae, (Lethrinae, Geotrupinae)) and establishing a sister-group relationship between *Anoplotrupes* and *Geotrupes* within Geotrupinae. All PCGs were under strong purifying selection, and no widespread positive selection linked to high-altitude adaptation was detected. Together, these results provide a crucial phylogenetic framework and genomic resources for the family. Future studies should integrate nuclear genomic data and focus on the functional role of dynamic non-coding regions to fully unravel the group’s evolutionary history and adaptation mechanisms.

## Data Availability

The two mitochondrial genome sequences newly sequenced in this study have been deposited at GenBank under the accession number PX715248-49.

## Supplementary Materials

The following supporting information can be downloaded at: https://www.mdpi.com/article/10.3390/biology15020164/s1, Figure S1: Size comparison of mitochondrial genomic components across Geotrupidae. Bars represent the lengths (in base pairs) of protein-coding genes (PCGs, concatenated), ribosomal RNA genes (*rrnL*, *rrnS*), the control region (CR), and transfer RNA genes (tRNAs, concatenated) for each species. Species are abbreviated as follows: As, *Anoplotrupes stercorosus* JX412838; Ast, *Anoplotrupes stercorosus* MN122896; Aste, *Anoplotrupes stercorosus* MT862428; Bo, *Bolboceratex* JX412746; Gs, *Geotrupes spiniger*; Gst, *Geotrupes stercorarius*; La, *Lethrus apterus*; Ls, *Lethrus scoparius*; Pa, *Phelotrupes auratus*; Po, *Phelotrupes oberthuri*; Figure S2: A+T content variation in mitochondrial protein-coding genes across Geotrupidae subfamilies. The scatter plot shows the mean A+T content (with standard deviation) for all 13 PCGs combined within each of the three subfamilies: Geotrupinae, Lethrinae, and Bolboceratinae; Figure S3: Nucleotide diversity varies among 13 protein-coding genes: (A) inter-gene variation; (B) inter-genus variation. * *p* < 0.05, ** *p* < 0.01, *** *p* < 0.001; independent samples *t*-test. Different colors denote different genera; Figure S4: Nucleotide diversity (*Pi*) of mitochondrial protein-coding genes. Sliding window analysis (window: 100 bp, step: 25 bp) was performed separately for the two newly sequenced species (blue lines) and the eight previously published species (red lines) to compare sequence polymorphism across each of the 13 PCGs; Figure S5: Evolutionary rates and GC content of mitochondrial protein-coding genes in Geotrupidae. For each of the 13 PCGs, the bar chart shows the mean *Ka*/*Ks* ratio (left Y-axis) across all species, and the line plot shows the corresponding mean G+C content (right Y-axis). Error bars represent standard deviation; Figure S6: Correlation between evolutionary rate (*Ka*/*Ks*) and G+C content for mitochondrial protein-coding genes. Each point represents one of the 13 PCGs. The regression line and coefficient of determination (*R*^2^) are shown; Figure S7: Relative Synonymous Codon Usage (RSCU) in the two newly sequenced mitogenomes. RSCU values for all codons used in the mitochondrial PCGs of *Geotrupes stercorarius* and *Phelotrupes auratus*. Codon families are color-coded by their corresponding amino acid. An RSCU value > 1.0 indicates a codon is used more frequently than expected under equal usage; Figure S8: Patterns of codon usage in Geotrupidae mitogenomes. (A) Relationship between the effective number of codons (ENC) and GC3 content. The solid curve (ENC*) represents the expected ENC under pure mutational bias. (B) Relationship between GC content at the first and second codon positions (GC12) and at the third position (GC3). The diagonal dashed line (y = x) is provided for reference; Figure S9: Structural organization of the intergenic spacer between *nad2* and *trnW* in Geotrupidae mitogenomes. The diagram annotations follow the same scheme as in Figure 5; Figure S10: Sequence alignment of the conserved intergenic spacer between *trnS2* and *nad1*. Bases conserved across all ten Geotrupidae species are highlighted in dark gray. Sequences from the two newly sequenced species are marked with a red asterisk. Light blue shading indicates bases conserved within Geotrupinae, and light green shading indicates bases conserved within Lethrinae; Table S1: Sample collection details for the newly sequenced Geotrupidae specimens; Table S2: List of Geotrupidae species used in this study with their taxonomic classification and GenBank accession numbers. Newly sequenced mitogenomes are highlighted with an asterisk; Table S3: Results of substitution saturation tests (DAMBE analysis) for the phylogenetic datasets. Iss, index of substitution saturation; Iss.cS, the critical Iss value; Table S4: The best partitioning schemes and substitution models selected by IQ-TREE for the three datasets; Table S5: Annotation of the complete mitochondrial genomes of *Geotrupes stercorarius* and *Phelotrupes auratus*; Table S6: Results of the branch-site model in PAML; Table S7: Codon usage statistics for the 13 mitochondrial protein-coding genes across Geotrupidae mitogenomes. RSCU, relative synonymous codon usage; %, percentage of the total count; AA, amino acid; Table S8: Maximum tandem repeats in the mitochondrial control regions of nine Geotrupidae species; Table S9: Maximum tandem repeats in the mitochondrial “*nad2*-*trnW*” intergenic spacer of six Geotrupidae species. Reference [10] is cited in the Supplementary Materials.
