# Supplementary material for: Comparative Mitogenomics and Phylogeny of Geotrupidae (Insecta: Coleoptera): Insights from Two New Mitogenomes of Qinghai–Tibetan Plateau Dung Beetles
Source: Biology (Basel). 2026 Jan 16;15(2):164. doi: 10.3390/biology15020164 (PMC12838160; doi:10.3390/biology15020164)
Supplement: Supplementary file 1 [file biology-15-00164-s001.zip › biology-4083722-supplementary/Figure S10 Non-coding region between trnS2 and nad1.pdf]

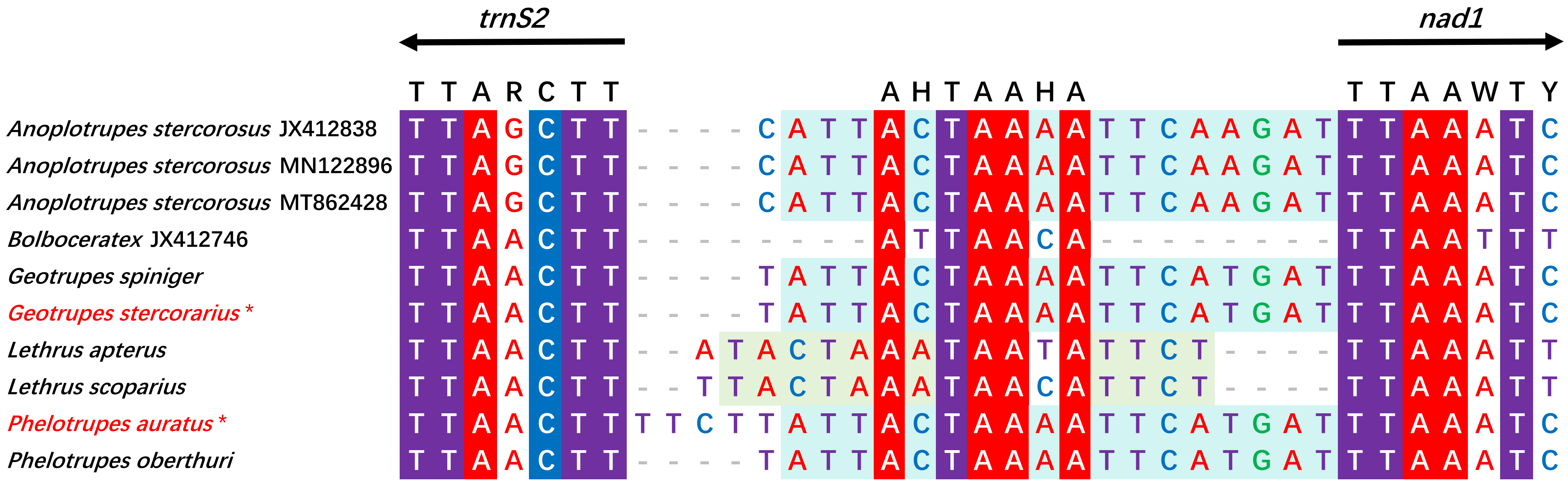
trnS2
nad1
T T A R C T T A H T A A H A T T A A W T Y
Anoplotrupes stercorosus JX412838
Anoplotrupes stercorosus MN122896
Anoplotrupes stercorosus MT862428
Bolboceratex JX412746
Geotrupes spiniger
Geotrupes stercorarius *
Lethrus apterus
Lethrus scoparius
Phelotrupes auratus *
Phelotrupes oberthuri
T T A G C T T - - - - C A T T A C T A A A A T T C A A G A T T T A A A T C
T T A G C T T - - - - C A T T A C T A A A A T T C A A G A T T T A A A T C
T T A G C T T - - - - C A T T A C T A A A A T T C A A G A T T T A A A T C
T T A A C T T - - - - - - - - A T T A A C A - - - - - - - - T T A A T T T
T T A A C T T - - - - T A T T A C T A A A A T T C A T G A T T T A A A T C
T T A A C T T - - - - T A T T A C T A A A A T T C A T G A T T T A A A T C
T T A A C T T - - A T A C T A A A T A A T A T T C T - - - - T T A A A T T
T T A A C T T - - T T A C T A A A T A A C A T T C T - - - - T T A A A T T
T T A A C T T T T C T T A T T A C T A A A A T T C A T G A T T T A A A T C
T T A A C T T - - - - T A T T A C T A A A A T T C A T G A T T T A A A T C
