# Supplementary material for: Comparative Mitogenomics and Phylogeny of Geotrupidae (Insecta: Coleoptera): Insights from Two New Mitogenomes of Qinghai–Tibetan Plateau Dung Beetles
Source: Biology (Basel). 2026 Jan 16;15(2):164. doi: 10.3390/biology15020164 (PMC12838160; doi:10.3390/biology15020164)
Supplement: Supplementary file 1 [file biology-15-00164-s001.zip › biology-4083722-supplementary/Figure S9 Sequence arrangement of the nad2 and trnW intergenic regions.pdf]

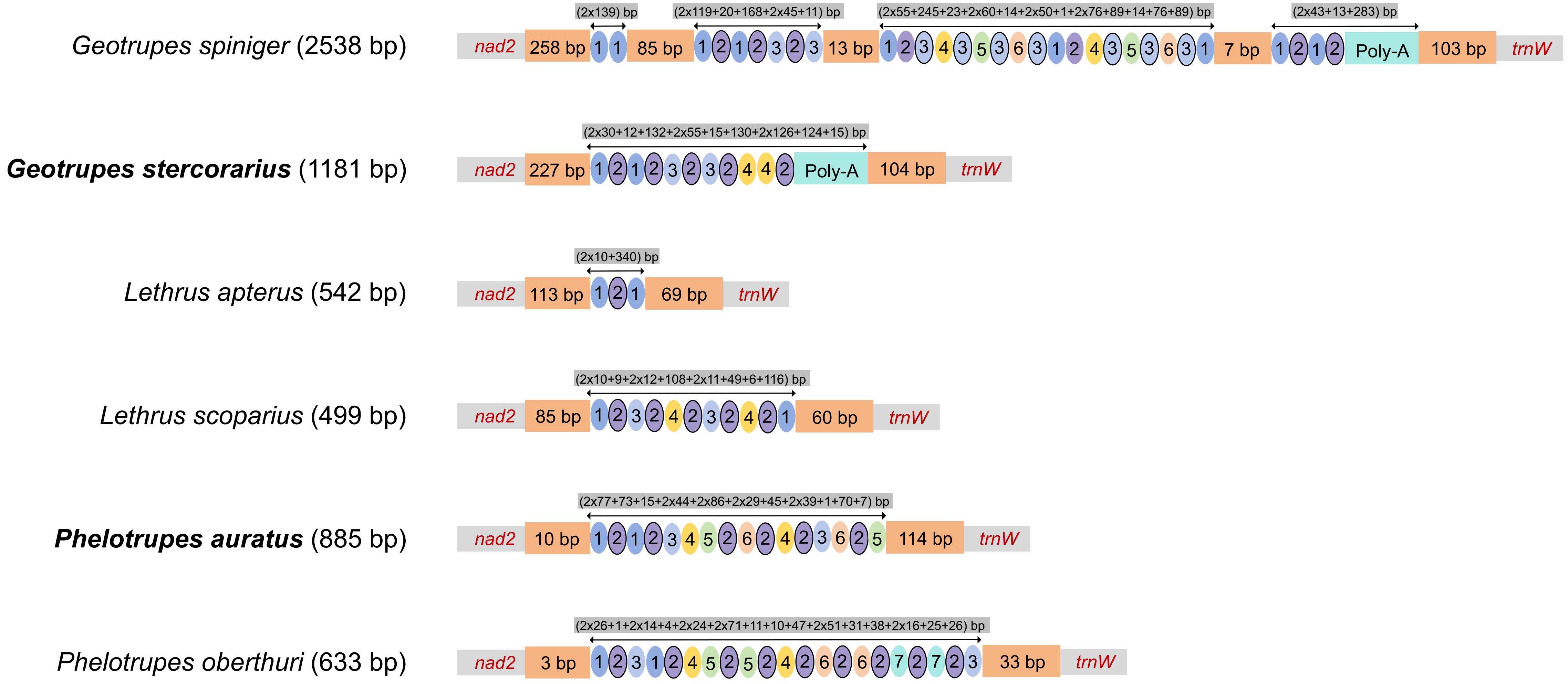

Geotrupes spiniger (2538 bp)
nad2
258 bp
(2x139) bp
1 1
85 bp
(2x119+20+168+2x45+11) bp
1 2 1 2 3 2 3
13 bp
(2x55+245+23+2x60+14+2x50+1+2x76+89+14+76+89) bp
1 2 3 4 3 5 3 6 3 1 2 4 3 5 3 6 3 1
7 bp
(2x43+13+283) bp
1 2 1 2
Poly-A
103 bp
trnW
Geotrupes stercorarius (1181 bp)
nad2
227 bp
(2x30+12+132+2x55+15+130+2x126+124+15) bp
1 2 1 2 3 2 3 2 4 4 2
Poly-A
104 bp
trnW
Lethrus apterus (542 bp)
nad2
113 bp
(2x10+340) bp
1 2 1
69 bp
trnW
Lethrus scoparius (499 bp)
nad2
85 bp
(2x10+9+2x12+108+2x11+49+6+116) bp
1 2 3 2 4 2 3 2 4 2 1
60 bp
trnW
Phelotrupes auratus (885 bp)
nad2
10 bp
(2x77+73+15+2x44+2x86+2x29+45+2x39+1+70+7) bp
1 2 1 2 3 4 5 2 6 2 4 2 3 6 2 5
114 bp
trnW
Phelotrupes oberthuri (633 bp)
nad2
3 bp
(2x26+1+2x14+4+2x24+2x71+11+10+47+2x51+31+38+2x16+25+26) bp
1 2 3 1 2 4 5 2 5 2 4 2 6 2 6 2 7 2 7 2 3
33 bp
trnW
