# Supplementary material for: Comparative Mitogenomics and Phylogeny of Geotrupidae (Insecta: Coleoptera): Insights from Two New Mitogenomes of Qinghai–Tibetan Plateau Dung Beetles
Source: Biology (Basel). 2026 Jan 16;15(2):164. doi: 10.3390/biology15020164 (PMC12838160; doi:10.3390/biology15020164)
Supplement: Supplementary file 1 [file biology-15-00164-s001.zip › biology-4083722-supplementary/Table S1 Details sampling information in this study.pdf]

Table S1 Details sampling information in this study.

Species	Sampling locality	Specimen voucher	Coordinate	Altitude (m)
Geotrupes stercorarius	Yushu County, Qinghai Province, China	GYSJGN	33°01′N, 97°30′E	3841
Phelotrupes auratus	Menyuan County, Qinghai Province, China	CMYHCO	37°37′N, 101°22′E	3400
