# Supplementary material for: Comparative Mitogenomics and Phylogeny of Geotrupidae (Insecta: Coleoptera): Insights from Two New Mitogenomes of Qinghai–Tibetan Plateau Dung Beetles
Source: Biology (Basel). 2026 Jan 16;15(2):164. doi: 10.3390/biology15020164 (PMC12838160; doi:10.3390/biology15020164)
Supplement: Supplementary file 1 [file biology-15-00164-s001.zip › biology-4083722-supplementary/Table S2 Information of Geotrupidae species used in this study.pdf]

Table S2 Information of Geotrupidae species used in this study and their taxonomic ranks. Newly sequenced mitogenomes are highlighted with an asterisk (*). The "-" means that we cannot find its reference in both NCBI website and internet.

Famliy	Subfamliy	Species	Accession Number	Reference
Geotrupidae	Bolboceratinae	*Bolboceratex* JX412746	JX412746	-
	Geotrupinae	*Anoplotrupes stercorosus* JX412838	JX412838	-
		Anoplotrupes stercorosus MN122896	MN122896	-
		Anoplotrupes stercorosus MT862428	MT862428	-
		Geotrupes spiniger	OY390743	-
		*Geotrupes stercorarius**	PX715248	This study
		*Phelotrupes auratus**	PX715249	This study
		Phelotrupes oberthuri	MT548773	-
	Lethrinae	*Lethrus apterus*	BK067253	-
		Lethrus scoparius	BK068120	Bubán, *et al.*, 2025
