# Supplementary material for: Comparative Mitogenomics and Phylogeny of Geotrupidae (Insecta: Coleoptera): Insights from Two New Mitogenomes of Qinghai–Tibetan Plateau Dung Beetles
Source: Biology (Basel). 2026 Jan 16;15(2):164. doi: 10.3390/biology15020164 (PMC12838160; doi:10.3390/biology15020164)
Supplement: Supplementary file 1 [file biology-15-00164-s001.zip › biology-4083722-supplementary/Table S3 Saturation Test by DAMBE.pdf]

Table S3 Substitution saturation tests performed using DAMBE for each of the 13 protein-coding genes (PCGs), three positions in the tandem arrangement of the 13 PCGs, the sequence of the 13 PCG sequences tandemly arranged with two rRNAs, and the two rRNA genes. Iss, index of substitution saturation; Iss.cS, the critical Iss value.

Dataset	Iss	Iss.cS	*P* value
atp6	0.2800	0.7418	＜0.0001
atp8	0.4003	0.6796	＜0.0001
cob	0.2562	0.7737	＜0.0001
cox1	0.3100	0.7899	＜0.0001
cox2	0.2650	0.7432	＜0.0001
cox3	0.2343	0.7517	＜0.0001
nad1	0.2718	0.7633	＜0.0001
nad2	0.7231	0.7668	＜0.0001
nad3	0.3291	0.7007	＜0.0001
nad4	0.2611	0.7825	＜0.0001
nad4L	0.2638	0.6912	＜0.0001
nad5	0.2779	0.7956	＜0.0001
nad6	0.4389	0.7214	＜0.0001
P123	0.3217	0.8356	＜0.0001
Position 1st	0.2617	0.8257	＜0.0001
Position 2nd	0.1601	0.8257	＜0.0001
Position 3rd	0.6178	0.8257	＜0.0001
P123R	0.3738	0.8362	＜0.0001
rrnS	0.6750	0.7535	＜0.0001
rrnL	0.6476	0.7848	＜0.0001
