# Supplementary material for: Comparative Mitogenomics and Phylogeny of Geotrupidae (Insecta: Coleoptera): Insights from Two New Mitogenomes of Qinghai–Tibetan Plateau Dung Beetles
Source: Biology (Basel). 2026 Jan 16;15(2):164. doi: 10.3390/biology15020164 (PMC12838160; doi:10.3390/biology15020164)
Supplement: Supplementary file 1 [file biology-15-00164-s001.zip › biology-4083722-supplementary/Table S4 The best partitioning schemes and substitution models selected.pdf]

Table S4 The best partitioning schemes and substitution models selected by IQ-TREE for the three datasets.

Dataset	Subset	Best-fit scheme	Model
P123	P1	*atp6*_pos1, *cob*_pos1, *cox1*_pos1, *cox2*_pos1, *cox3*_pos1	TIM2+F+I+G4
	P2	*atp6*_pos2, *cob*_pos2, *cox1*_pos2, *cox2*_pos2, *cox3*_pos2	K3Pu+F+I
	P3	*atp6*_pos3, *nad3*_pos3, *nad6*_pos3	HKY+F+I+G4
	P4	*atp8*_pos1, *atp8*_pos3, *nad4L*_pos3	HKY+F+G4
	P5	*atp8*_pos2, *nad6*_pos2	TPM3u+F+G4
	P6	*cob*_pos3, *cox1*_pos3, *cox2*_pos3, *cox3*_pos3	HKY+F+I+R2
	P7	*nad1*_pos1, *nad4*_pos1, *nad4L*_pos1, *nad5*_pos1	TIM2+F+I+G4
	P8	*nad1*_pos2, *nad2*_pos2, *nad3*_pos2, *nad4*_pos2, *nad4L*_pos2, *nad5*_pos2	TVM+F+R2
	P9	*nad1*_pos3, *nad4*_pos3	HKY+F+R2
	P10	*nad2*_pos1, *nad3*_pos1, *nad6*_pos1	TIM2+F+I+G4
	P11	*nad2*_pos3	HKY+F+R2
	P12	*nad5*_pos3	HKY+F+I+R2
P123R	P1	*atp6*_pos1, *cob*_pos1	TIM2+F+I+G4
	P2	*atp6*_pos2, *cob*_pos2, *cox1*_pos2, *cox2*_pos2, *cox3*_pos2	K3Pu+F+I
	P3	*atp6*_pos3, *nad2*_pos3, *nad3*_pos3, *nad6*_pos3	HKY+F+R2
	P4	*atp8*_pos1, *atp8*_pos3, *nad4L*_pos3	HKY+F+G4
	P5	*atp8*_pos2, *nad6*_pos2	TPM3u+F+G4
	P6	*cob*_pos3, *cox1*_pos3, *cox2*_pos3, *cox3*_pos3	TN+F+G4
	P7	*cox1*_pos1, *cox2*_pos1, *cox3*_pos1	TIM2+F+G4
	P8	*nad1*_pos1, *nad4*_pos1, *nad4L*_pos1, *nad5*_pos1	TIM2+F+I+G4
	P9	*nad1*_pos2, *nad2*_pos2, *nad3*_pos2, *nad4*_pos2, *nad4L*_pos2, *nad5*_pos2	TVM+F+R2

	P10	*nad1*_pos3, *nad4*_pos3	HKY+F+I+R2
	P11	*nad2*_pos1, *nad3*_pos1, *nad6*_pos1	TIM2+F+I+G4
	P12	*nad5*_pos3	HKY+F+I+R2
	P13	*rrnL*, *rrnS*	GTR+F+G4
P123AA	P1	*atp6*, *cob*, *cox1*, *cox2*, *cox3*, *nad1*, *nad4*, *nad4L*, *nad5*	MTART+R3
	P2	*atp8*, *nad2*, *nad3*, *nad6*	MTMET+F+I+R2
