# Supplementary material for: Comparative Mitogenomics and Phylogeny of Geotrupidae (Insecta: Coleoptera): Insights from Two New Mitogenomes of Qinghai–Tibetan Plateau Dung Beetles
Source: Biology (Basel). 2026 Jan 16;15(2):164. doi: 10.3390/biology15020164 (PMC12838160; doi:10.3390/biology15020164)
Supplement: Supplementary file 1 [file biology-15-00164-s001.zip › biology-4083722-supplementary/Table S5 Annotation of the two newly sequenced species mitochondrial genomes.pdf]

Table S5A Annotation of the *Geotrupes stercorarius* mitochondrial genome.

Name	Strand	Position		Length(bp)	Intergenic nucleotides(bp)	Codon		
						Anti	Start	Stop
nad2	+	1	1014	1014	0		ATG	TAA
trnW	+	2196	2263	68	1181	TCA		
trnC	-	2256	2316	61	-8	GCA		
trnY	-	2317	2380	64	0	GTA		
cox1	+	2380	3913	1534	-1		CTG	T
trnL2	+	3914	3978	65	0	TAA		
cox2	+	3978	4662	685	-1		ATG	T
trnK	+	4663	4734	72	0	CTT		
trnD	+	4734	4797	64	-1	GTC		
atp8	+	4798	4953	156	0		ATA	TAA
atp6	+	4947	5620	674	-7		ATG	TA
cox3	+	5621	6407	787	0		ATG	T
trnG	+	6408	6475	68	0	TCC		
nad3	+	6476	6827	352	0		ATT	T
trnA	+	6828	6891	64	0	TGC		
trnR	+	6891	6958	68	-1	TCG		
trnN	+	6960	7024	65	1	GTT		
trnS1	+	7025	7091	67	0	TCT		
trnE	+	7097	7160	64	5	TTC		
trnF	-	7159	7224	66	-2	GAA		
nad5	-	7225	8945	1721	0		ATT	TA
trnH	-	8946	9010	65	0	GTG		
nad4	-	9011	10347	1337	0		ATG	TA
nad4L	-	10341	10631	291	-7		ATG	TAA
trnT	+	10634	10695	62	2	TGT		
trnP	-	10696	10762	67	0	TGG		
nad6	+	10764	11266	503	1		ATC	TA
cob	+	11267	12407	1141	0		ATG	T
trnS2	+	12408	12472	65	0	TGA		
nad1	-	12492	13442	951	19		ATT	TAA
trnL1	-	13444	13506	63	1	TAG		
l-rRNA	-	13507	14807	1301	0			
trnV	-	14808	14877	70	0	TAC		
s-rRNA	-	14878	15664	787	0			
trnI	+	23309	23376	68	0	GAT		
trnQ	-	23377	23445	69	0	TTG		
trnM	+	23450	23518	69	4	CAT		

Table S5B Annotation of the *Phelotrupes auratus* mitochondrial genome.

Name	Strand	Position		Length(bp)	Intergenic nucleotides(bp)	Codon		
						Anti	Start	Stop
nad2	+	1	1014	1014	0		ATT	TAG
trnW	+	1900	1971	72	885	TCA		
trnC	-	1964	2025	62	-8	GCA		
trnY	-	2026	2090	65	0	GTA		
cox1	+	2090	3623	1534	-1		CTG	T
trnL2	+	3624	3688	65	0	TAA		
cox2	+	3688	4371	684	-1		ATG	TAA
trnK	+	4373	4444	72	1	CTT		
trnD	+	4444	4508	65	-1	GTC		
atp8	+	4509	4664	156	0		ATT	TAA
atp6	+	4658	5331	674	-7		ATG	TA
cox3	+	5332	6118	787	0		ATG	T
trnG	+	6119	6186	68	0	TCC		
nad3	+	6187	6538	352	0		ATT	T
trnA	+	6539	6603	65	0	TGC		
trnR	+	6603	6669	67	-1	TCG		
trnN	+	6672	6735	64	2	GTT		
trnS1	+	6736	6803	68	0	TCT		
trnE	+	6809	6872	64	5	TTC		
trnF	-	6871	6937	67	-2	GAA		
nad5	-	6939	8660	1722	1		ATT	TAA
trnH	-	8661	8726	66	0	GTG		
nad4	-	8727	10062	1336	0		ATG	TA
nad4L	-	10056	10346	291	-7		ATG	TAA
trnT	+	10349	10411	63	2	TGT		
trnP	-	10412	10477	66	0	TGG		
nad6	+	10479	10981	503	1		ATT	TA
cob	+	10982	12123	1142	0		ATG	TA
trnS2	+	12124	12189	66	0	TGA		
nad1	-	12213	13163	951	23		ATT	TAA
trnL1	-	13165	13231	67	1	TAG		
l-rRNA	-	13232	14531	1300	0			
trnV	-	14532	14601	70	0	TAC		
s-rRNA	-	14602	15392	791	0			
trnI	+	16482	16548	67	0	GAT		
trnQ	-	16553	16621	69	4	TTG		
trnM	+	16621	16689	69	-1	CAT		
