# Supplementary material for: Comparative Mitogenomics and Phylogeny of Geotrupidae (Insecta: Coleoptera): Insights from Two New Mitogenomes of Qinghai–Tibetan Plateau Dung Beetles
Source: Biology (Basel). 2026 Jan 16;15(2):164. doi: 10.3390/biology15020164 (PMC12838160; doi:10.3390/biology15020164)
Supplement: Supplementary file 1 [file biology-15-00164-s001.zip › biology-4083722-supplementary/Table S6 Results of the branch-site model in PAML.pdf]

Table S6 Results of the branch-site model in PAML.

Forebranch	Gene	Model	Likelihood (*L*)	Site class	0	1	2a	2b	2Δ*L*	*P*	Positively selected sites
Phelotrupes auratus	*atp6*	Model A (Null)	-961.9	proportion	0.4997	0.0900	0.3478	0.0626			
				background ω	0.0217	1.0000	0.0217	1.0000			
				foreground ω	0.0217	1.0000	1.0000	1.0000			
		Model A (Alternative)	-956.9	proportion	0.7517	0.1417	0.0896	0.0169			
				background ω	0.0223	1.0000	0.0223	1.0000			
				foreground ω	0.0223	1.0000	999.0000	999.0000	9.998868	0.0015664	15M 0.973*; 25M 0.997**; 26L 0.998**; 40N 0.969*; 4F 0.961*
Geotrupes stercorarius	*nad5*	Model A (Null)	-8714.4	proportion	0.9641	0.0263	0.0094	0.0003			
				background ω	0.0124	1.0000	0.0124	1.0000			
				foreground ω	0.0124	1.0000	1.0000	1.0000			
		Model A (Alternative)	-8712.5	proportion	0.9656	0.0267	0.0075	0.0002			
				background ω	0.0126	1.0000	0.0126	1.0000			
				foreground ω	0.0126	1.0000	45.9122	45.9122	3.80427	0.0511221	184I 0.955*
