# Supplementary material for: Comparative Mitogenomics and Phylogeny of Geotrupidae (Insecta: Coleoptera): Insights from Two New Mitogenomes of Qinghai–Tibetan Plateau Dung Beetles
Source: Biology (Basel). 2026 Jan 16;15(2):164. doi: 10.3390/biology15020164 (PMC12838160; doi:10.3390/biology15020164)
Supplement: Supplementary file 1 [file biology-15-00164-s001.zip › biology-4083722-supplementary/Table S8 Maximum tandem repeats in control regions of Geotrupidae mitogenomes.pdf]

Table S8 Maximum tandem repeats in control regions of Geotrupidae mitogenomes.

Species	Repeat sequence	Length	Perfect repetition
Anoplotrupes stercorosus JX412838	AGGACAAATA	10	2
Anoplotrupes stercorosus MN122896	TTATACAC	8	2
Anoplotrupes stercorosus MT862428	TTTATAGAT	9	2
Geotrupes spiniger	TTATTTTCAAAAAGCTTGGTGCCCGGCTACCAGCCTAAAAAATATATAAAAATTAAAGAAAA<br>ATTATTTTCAAAAAGCTTGGTGCCCGGCTACCAGCCTAAAAAATATATAAAAATTAAAGAAA<br>AATTATTTTCAAAAAGCTTGGTGCCCGGCTACCAGCCTAAAAAATATATAAAAATTAAA	183	2
*Geotrupes stercorarius**	ACATAAAACCATAAAATTTAGTAATTTTAACCTACCTCTACTCGAAACCTAATCATGCAAAGT<br>ACCCTCTCCACTAAATTACTAAAATTAATGATCATTAATAATTCCTAAACAAATATTTAACCC<br>CAATTTGGAAAAATTTTGCAAAAAATACCCCTTTTTAG	164	2
Lethrus apterus	TGCAACAGCTAGCTGCGCTTACATAATTATCACTCATACGCTAAACTTATAAATTTACTTGAT<br>CAAAGTTACTTATTCGTAATATGATGAAAT	93	2
Lethrus scoparius	TACAACTGTTAGCTGTGCTTTACACAATTGTTATCAATACGTCAAATTCAAGAATAACATTCA<br>ATCAAAATTCAAATTTATCTCTCATACGTATTGCATAATTGTTCAAGTACAACTGTTAGCTGTG<br>CTTTACACAATTATTATCAATACGTCAAATTCAAGAATAGCATTTGATCAAAATCCAAATTTA<br>TCTCTCATACGTATTACATAATTGTTCAAGTACAACTGTTAGCTGTGCTTTACACAATTGATAT<br>CAATACGTCAAATTCAAGAATAGCA	279	2
*Phelotrupes auratus**	TACTAAAATTTATAATCATGGAA	23	2
Phelotrupes oberthuri	CTAATTAATATTACCTTGAAAATTGAATGAAAAAGCTTGGTGCCCGGCCCTAGCCTATAATAA<br>TTATACACATAGGATAATATTACTATAATTAATTATTAATTTTACAAAATTAATT	118	2

Newly sequenced mitogenomes are highlighted with an asterisk (*).
