# Supplementary material for: Comparative Mitogenomics and Phylogeny of Geotrupidae (Insecta: Coleoptera): Insights from Two New Mitogenomes of Qinghai–Tibetan Plateau Dung Beetles
Source: Biology (Basel). 2026 Jan 16;15(2):164. doi: 10.3390/biology15020164 (PMC12838160; doi:10.3390/biology15020164)
Supplement: Supplementary file 1 [file biology-15-00164-s001.zip › biology-4083722-supplementary/Table S9 Maximum tandem repeats in nad2 and trnW of six Geotrupidae species.pdf]

Table S9 Maximum tandem repeats in *nad2* and *trnW* of six Geotrupidae species.

Species	Repeat sequence	Length	Perfect repetition
Geotrupes spiniger	TTTTAAAACCAATTTTAATTCAAAAAATTCTAAAATCAATTTTAATTCAAAAAATTTTAGAATAAATTTTAATTCAAAAAATTTTAAAATCAATTTTTAGATTAAAAAAAATTTAAAATCAATTTTTAGATTAAAAAAA	139	2
*Geotrupes stercorarius**	TTGAAATTAAGATTTGAAATTAAGATTTGAAATTAAGATTTGAAATTAAGATTTGAAATTAAGATTTGAAATTAAGATTTGAAATTAAGATTTGAAATTAAGATTTGAAATTAAGATTTGAAATTA	126	2
Lethrus apterus	TTAAGCCTTC	10	2
Lethrus scoparius	AAATTACTAATT	12	2
*Phelotrupes auratus**	AAAATACAATACTGCAAATTCAATTTAAAGTTATAATTTGAAAATACAATACTGCAAATTCAATTTAAAGTTATAATTTGAAAATA	84	2
Phelotrupes oberthuri	AAATTATAAAATTCTCATAAATCTAAATTTACATTTAATTGAAATTATAAAATTATAGCAATTTTAAATTC	71	2

Newly sequenced mitogenomes are highlighted with an asterisk (*).
