# Supplementary figures and images for: Comparative Mitogenomics and Phylogeny of Geotrupidae (Insecta: Coleoptera): Insights from Two New Mitogenomes of Qinghai–Tibetan Plateau Dung Beetles
Source: Biology (Basel). 2026 Jan 16;15(2):164. doi: 10.3390/biology15020164 (PMC12838160; doi:10.3390/biology15020164)

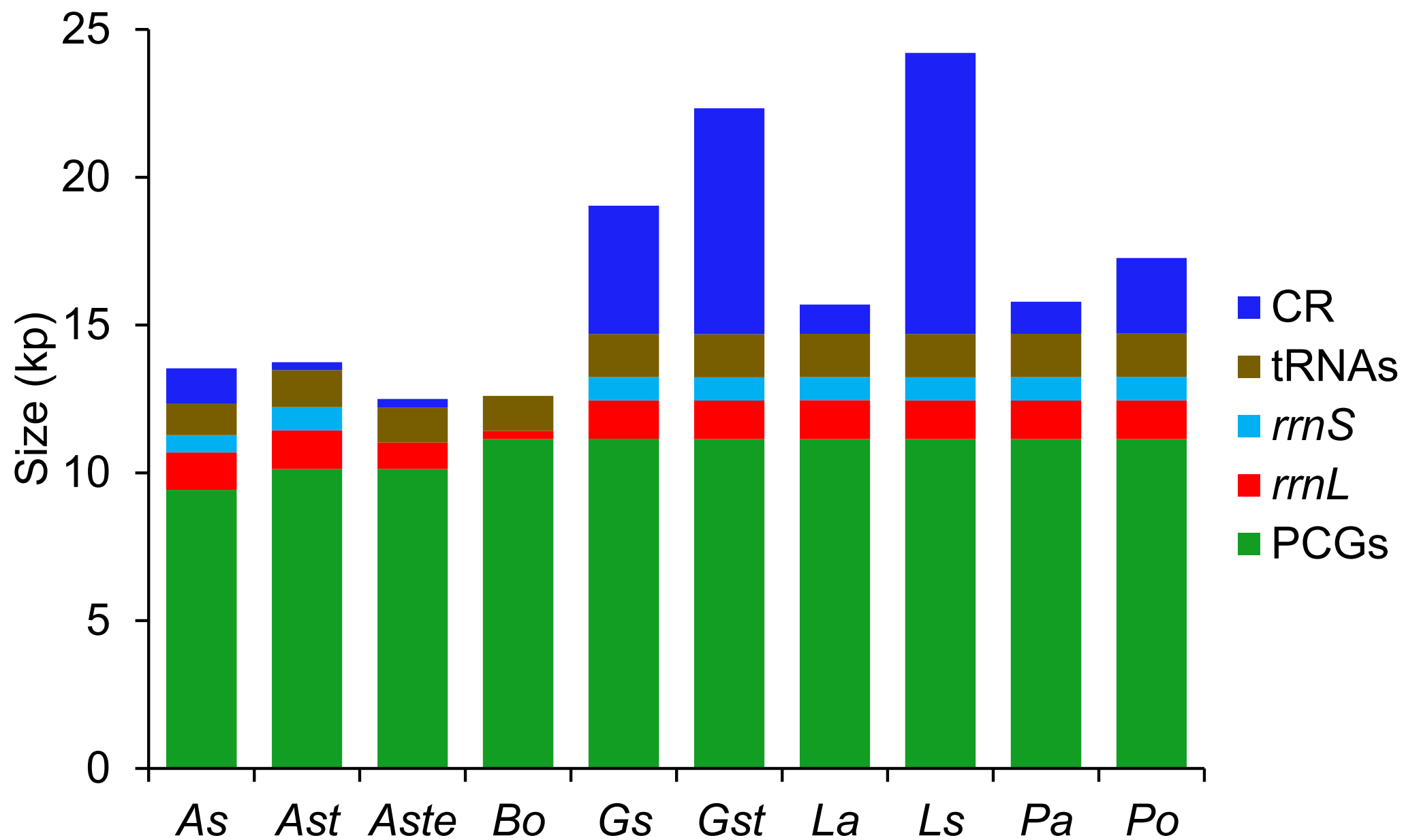

Supplement: Supplementary file 1 [file biology-15-00164-s001.zip › biology-4083722-supplementary/Figure S1 The size of PCGs, rrnL, rrnS, CR and tRNAs Geotrupidae mitochondrial genomes.pdf]

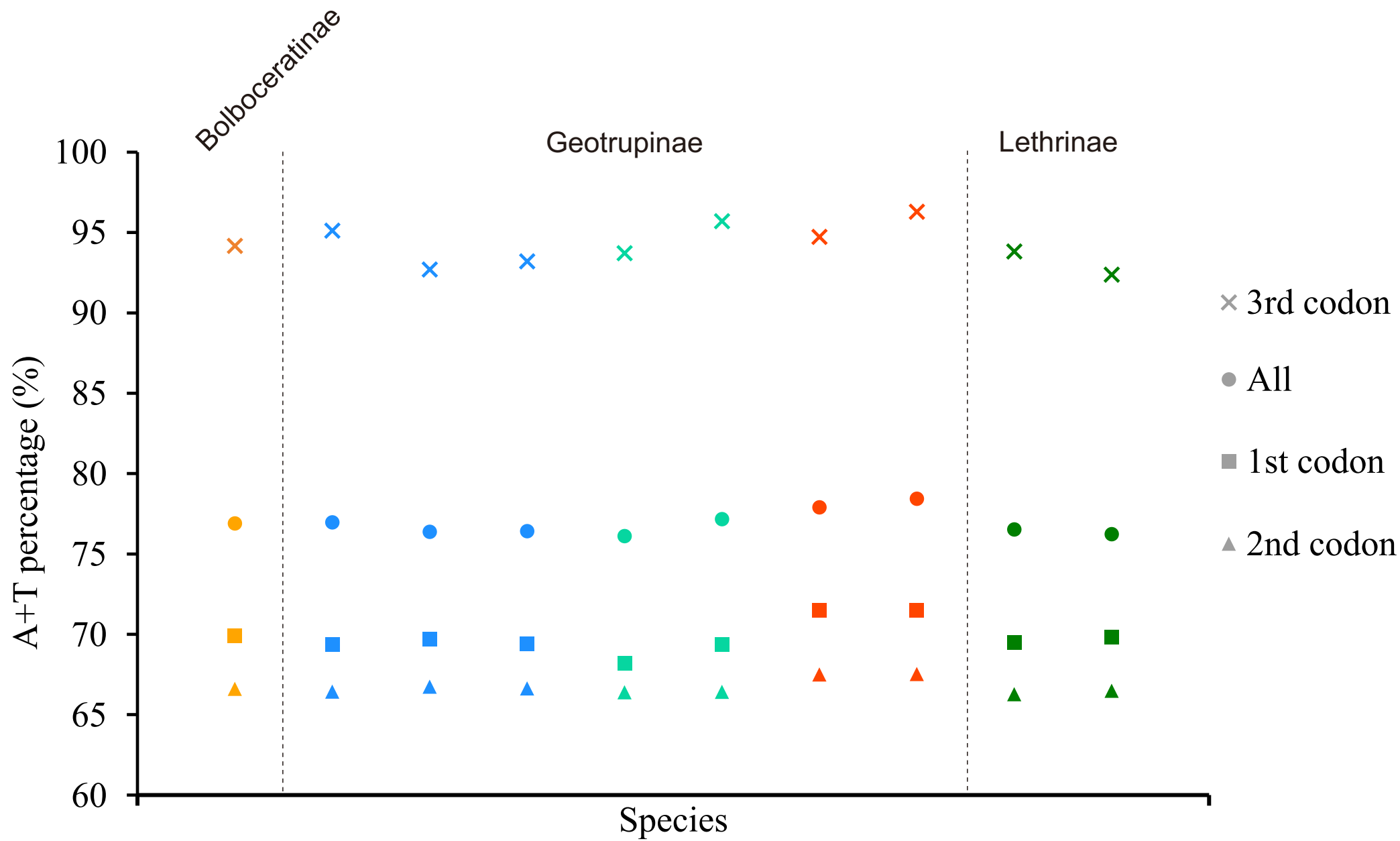

Supplement: Supplementary file 1 [file biology-15-00164-s001.zip › biology-4083722-supplementary/Figure S2 A + T% of the mitochondrial protein-coding genes among four serie.pdf]

A

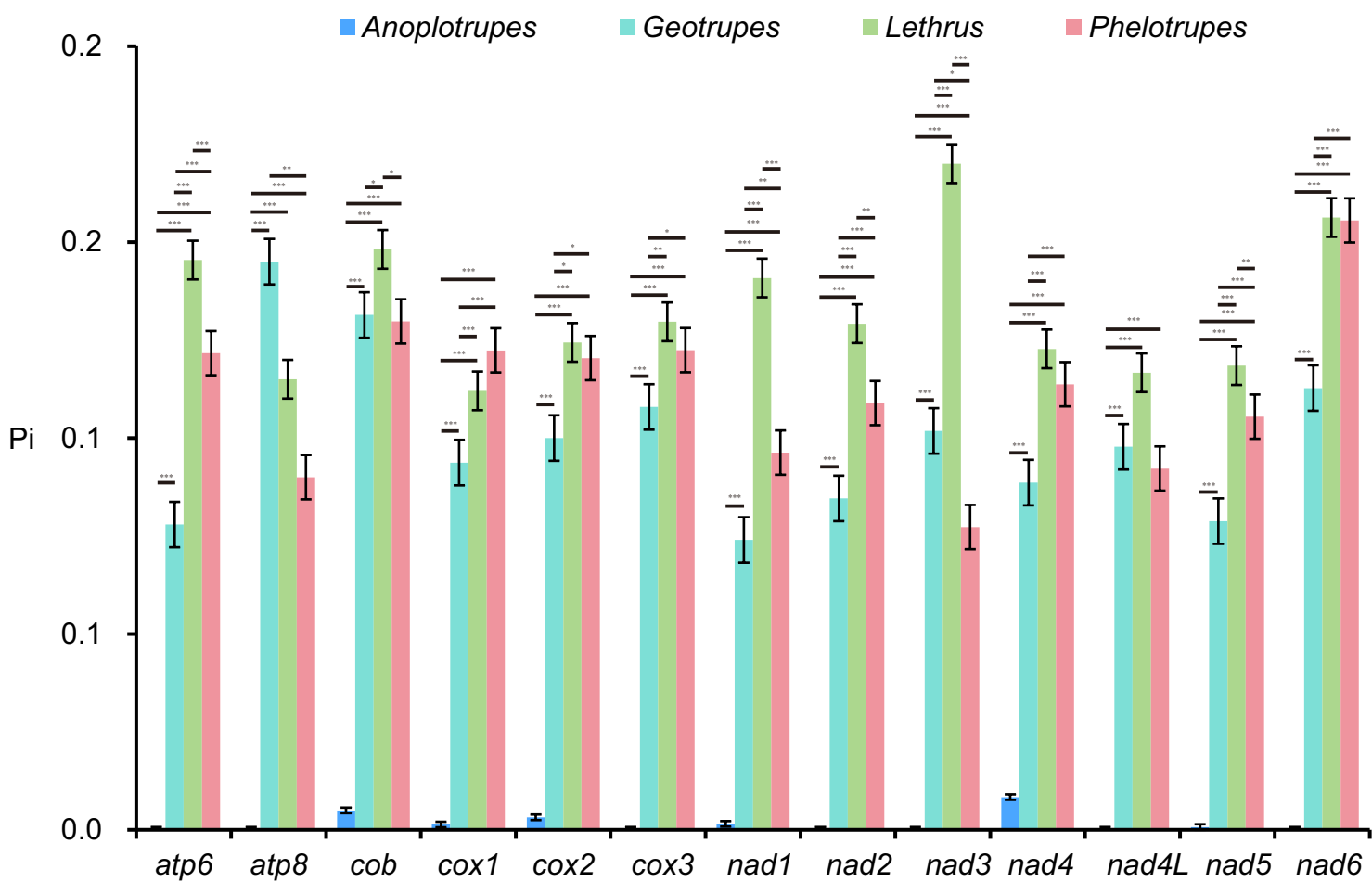

B

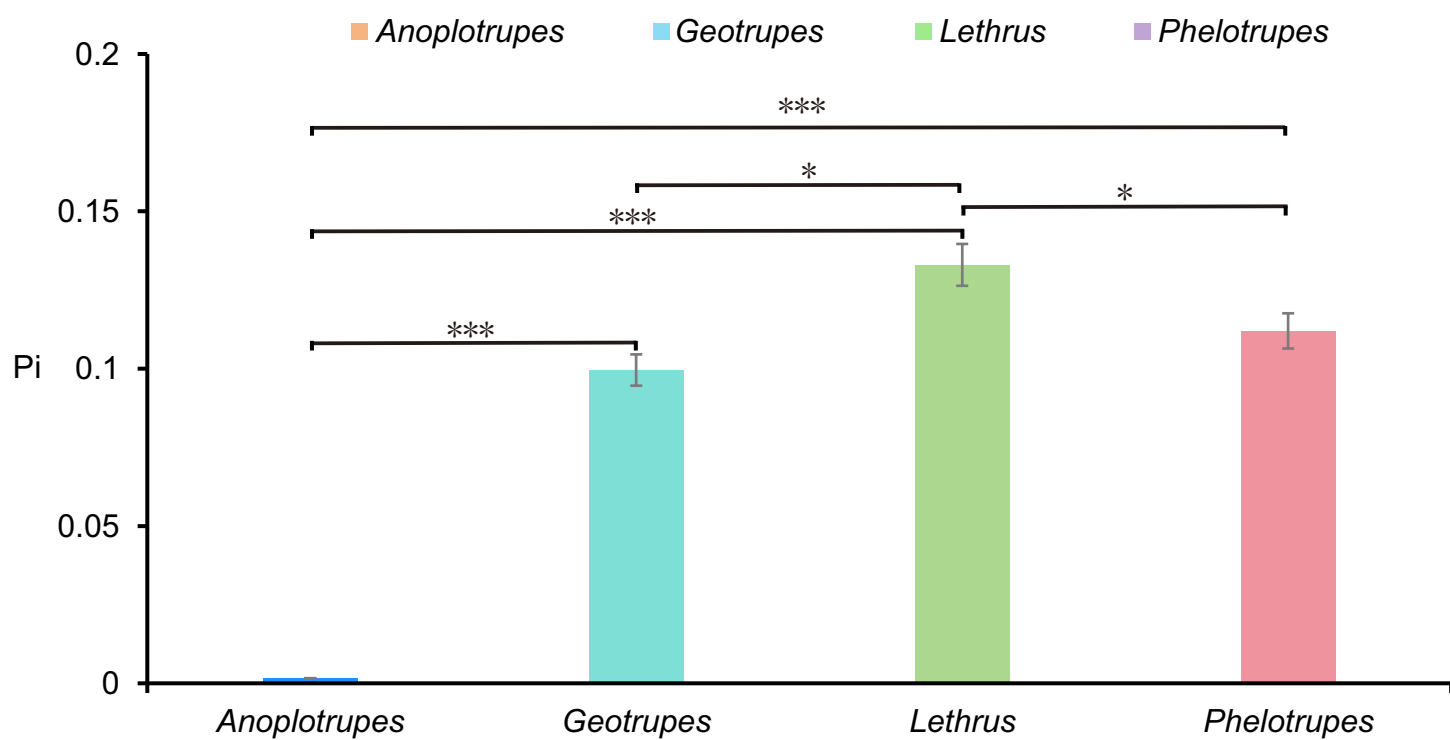

Supplement: Supplementary file 1 [file biology-15-00164-s001.zip › biology-4083722-supplementary/Figure S3 Nucleotide diversity of 13 PCGs differed across genera and individual genes.pdf]

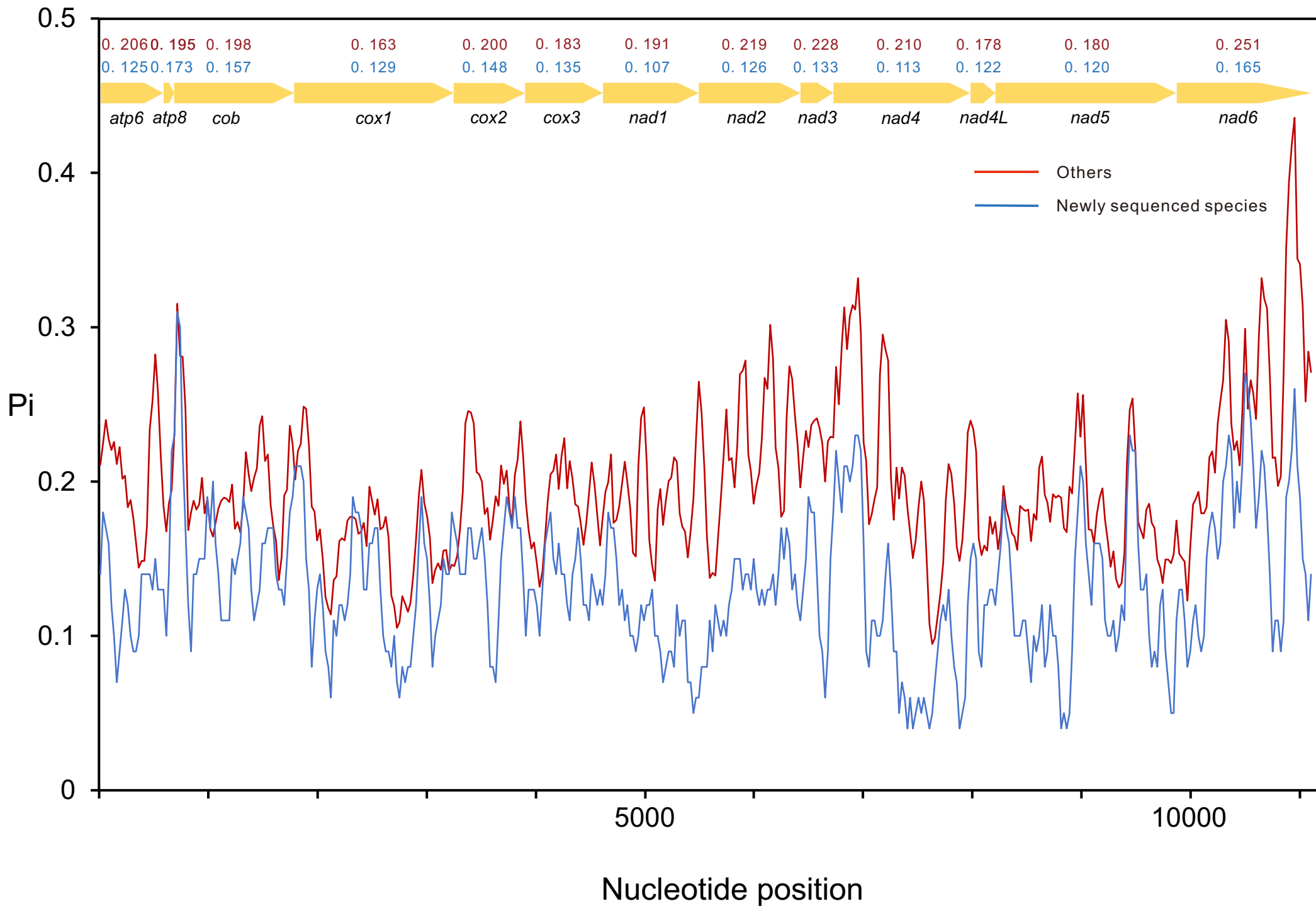

Supplement: Supplementary file 1 [file biology-15-00164-s001.zip › biology-4083722-supplementary/Figure S4 Nucleotide diversity (Pi) compared.pdf]

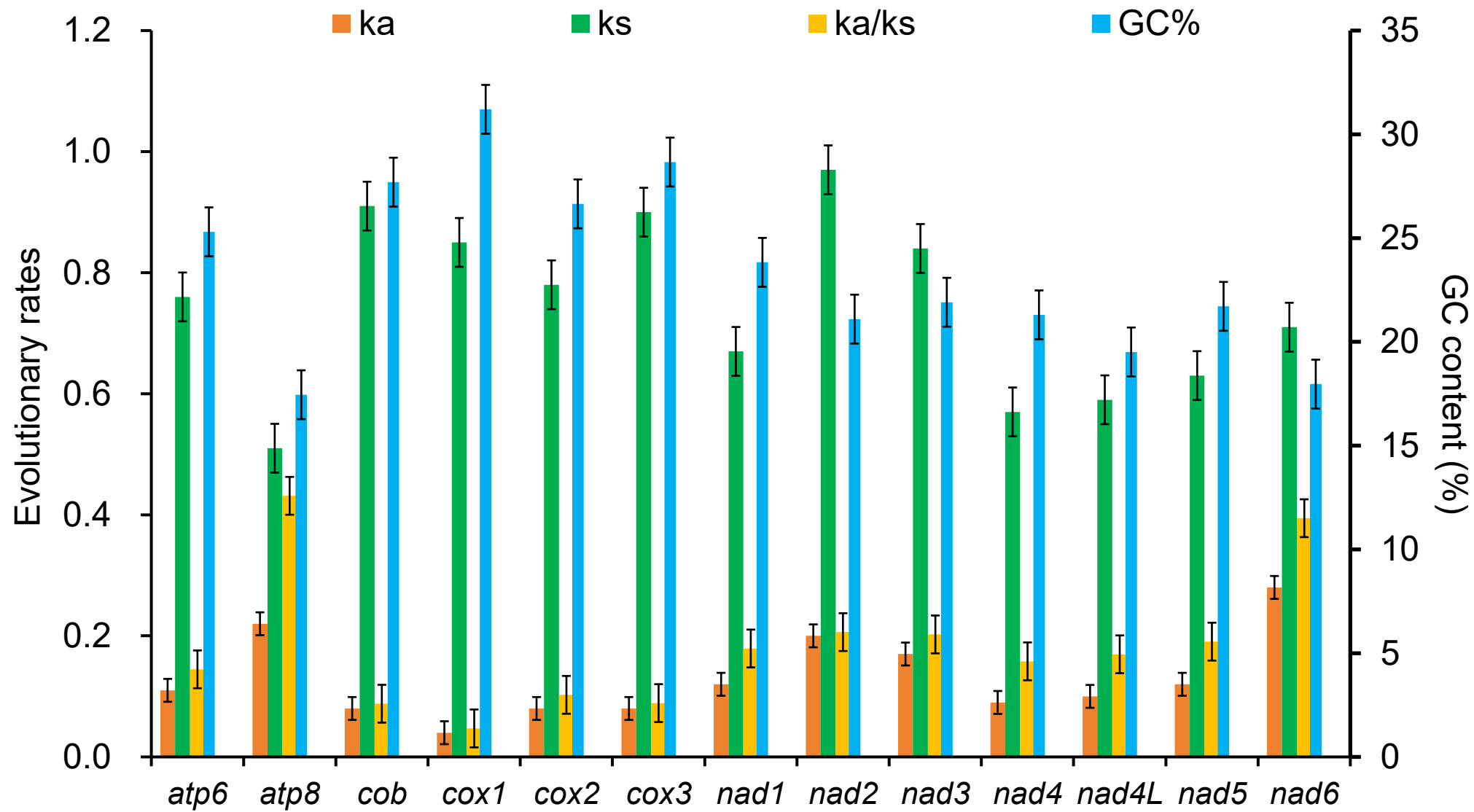

Supplement: Supplementary file 1 [file biology-15-00164-s001.zip › biology-4083722-supplementary/Figure S5 Evolutionary rates of 13 protein-coding genes.pdf]

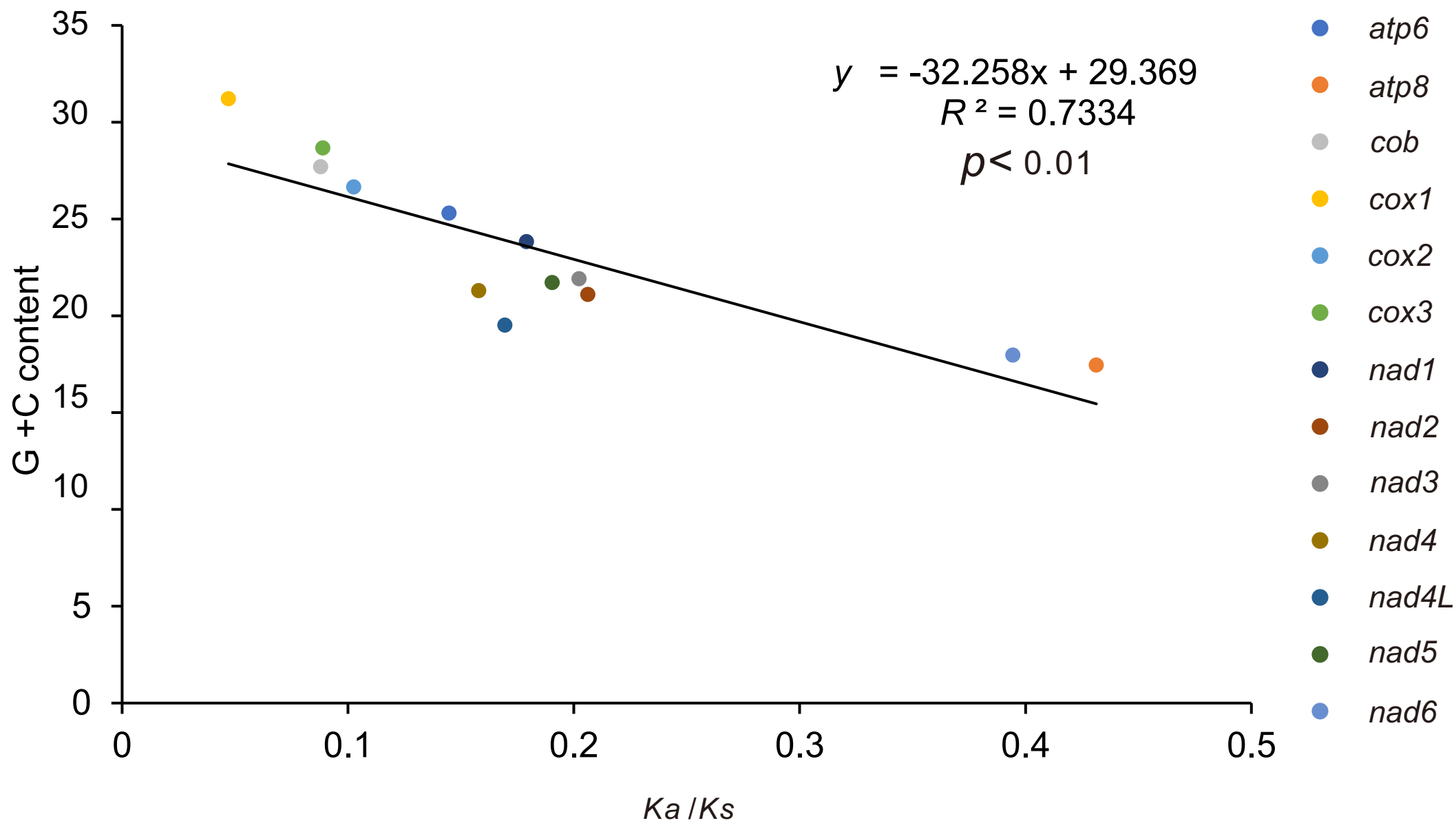

Supplement: Supplementary file 1 [file biology-15-00164-s001.zip › biology-4083722-supplementary/Figure S6 Correlation between evolutionary rate and G+C content.pdf]

A

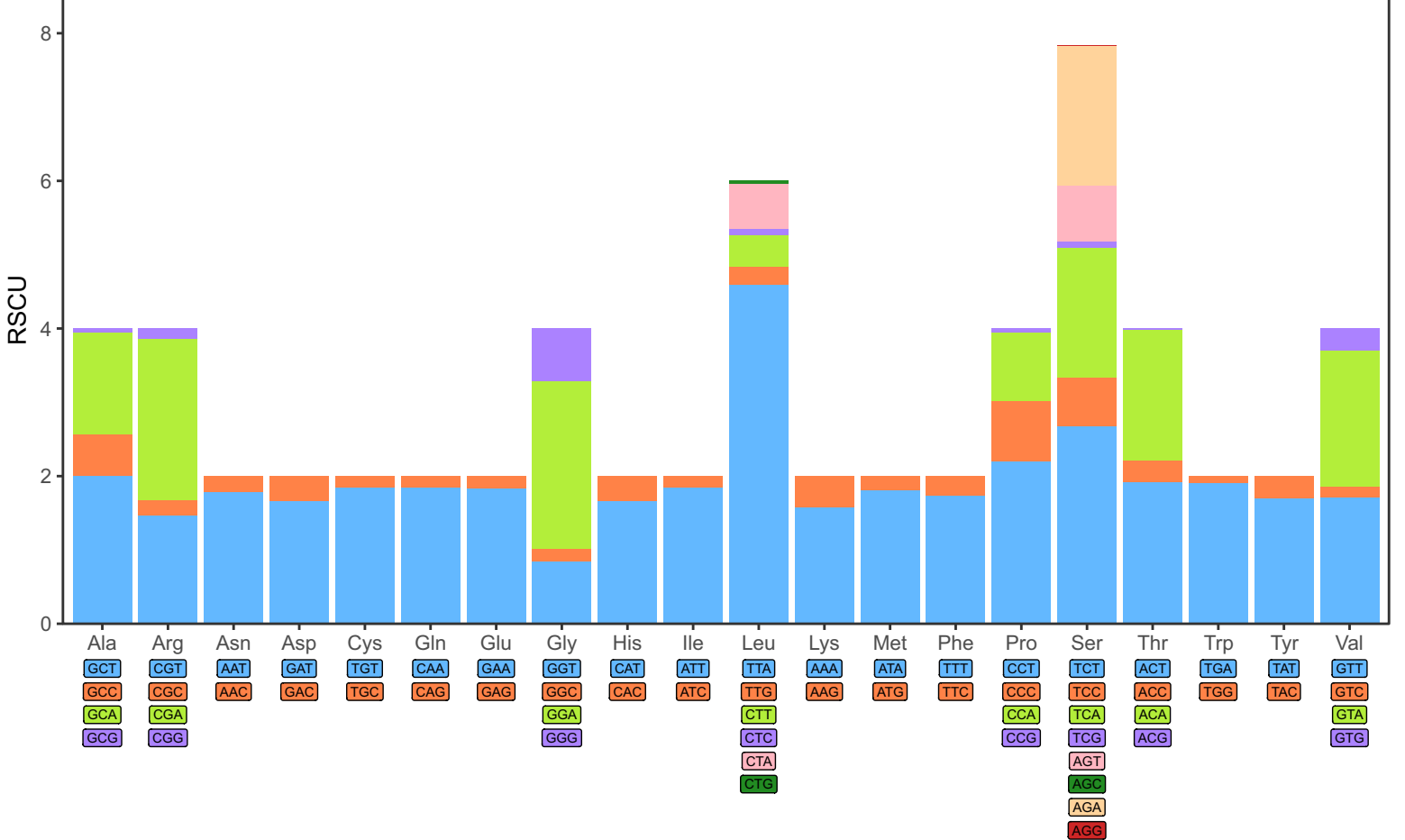

*Geotrupes stercorarius*

B

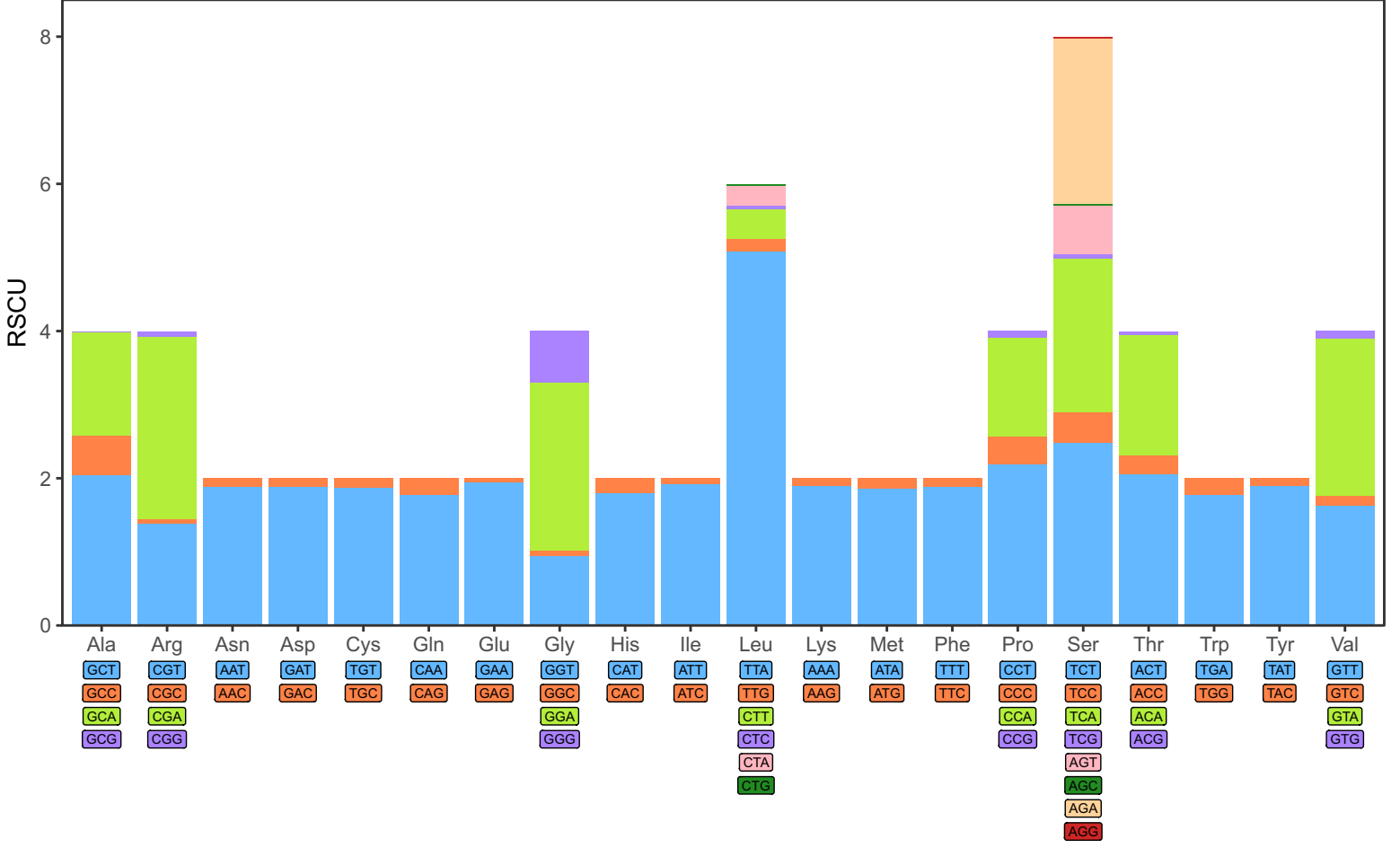

*Phelotrupes auratus*

Supplement: Supplementary file 1 [file biology-15-00164-s001.zip › biology-4083722-supplementary/Figure S7 Relative Synonymous Codon Usage (RSCU).pdf]

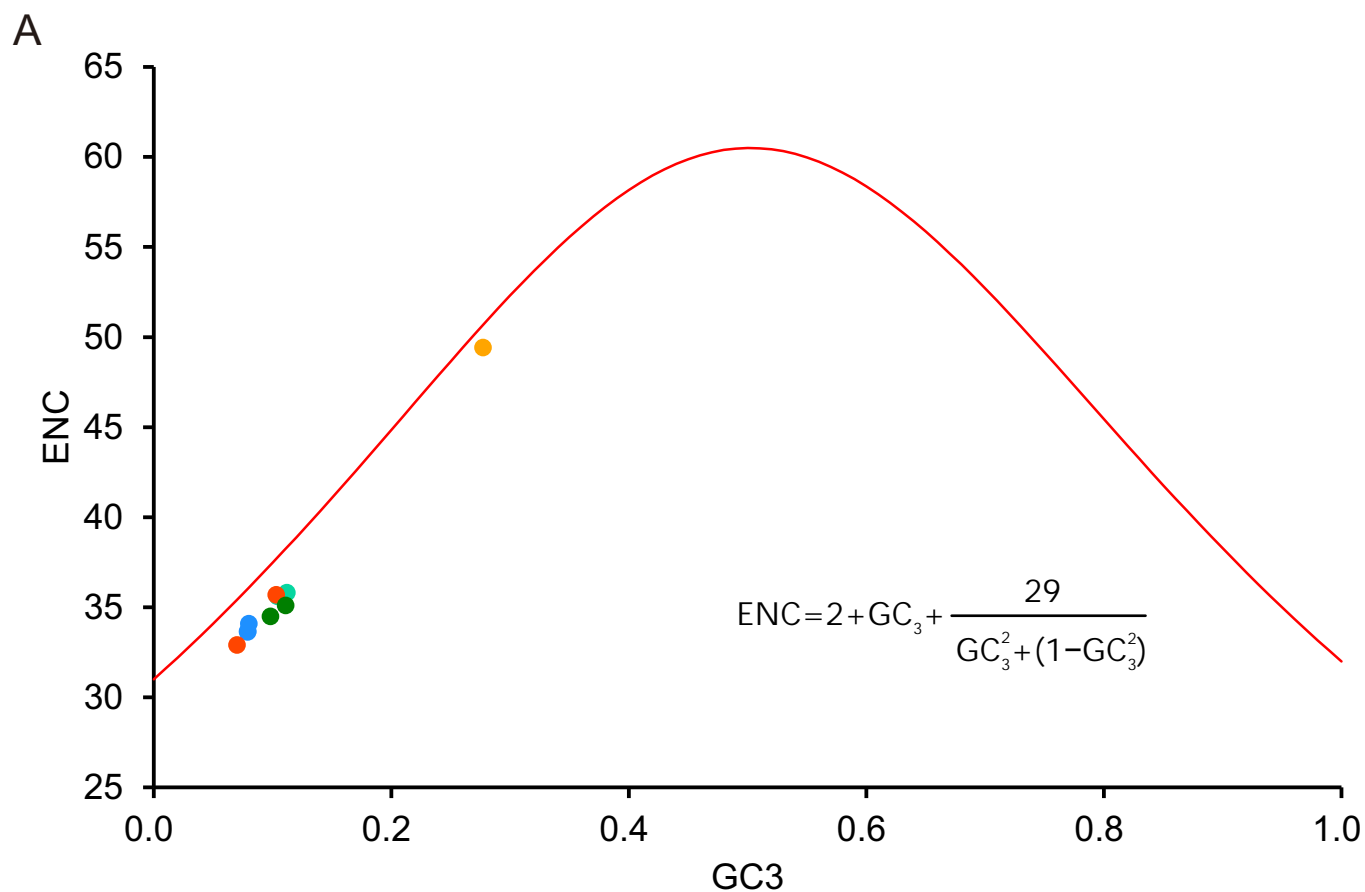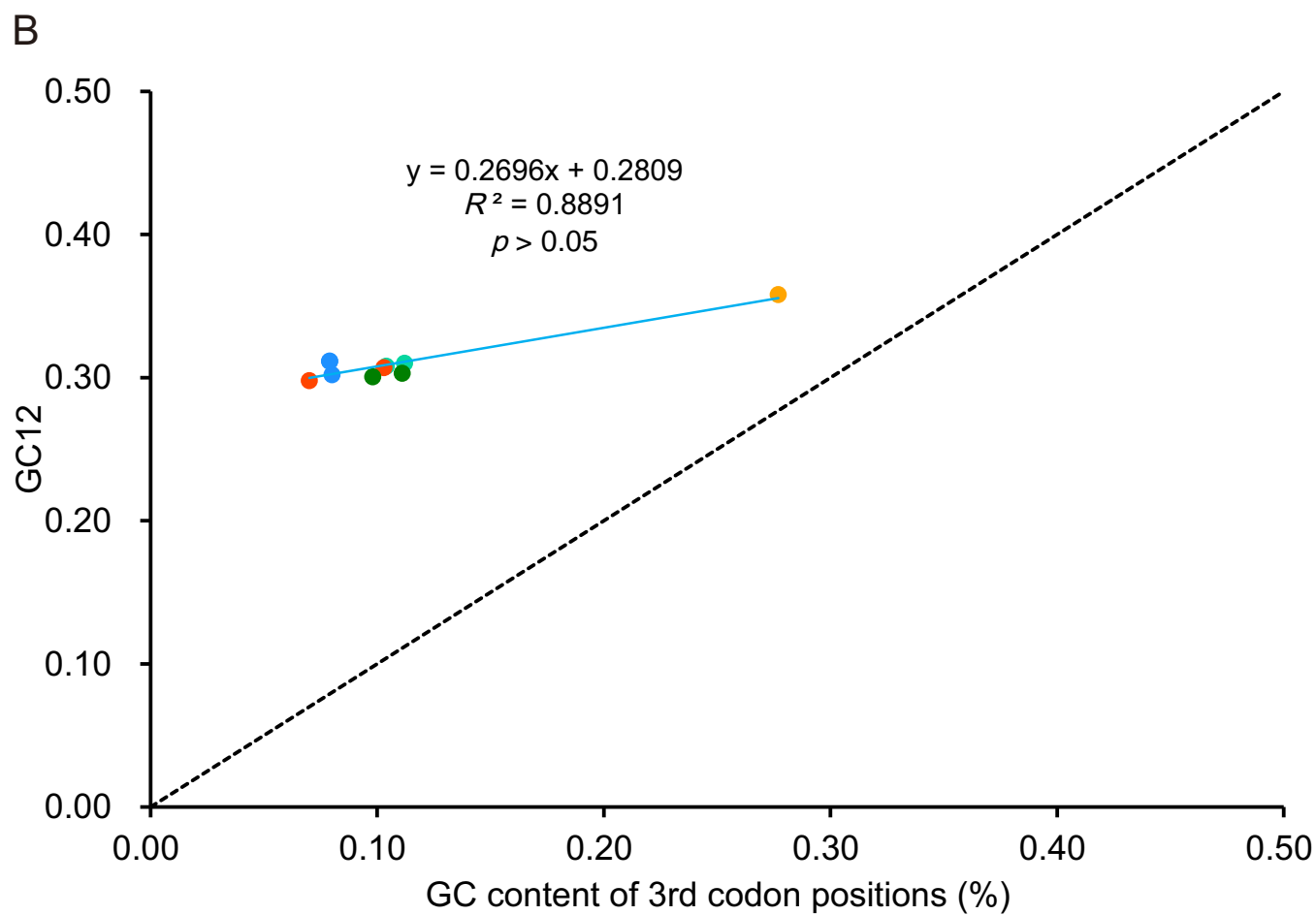

Supplement: Supplementary file 1 [file biology-15-00164-s001.zip › biology-4083722-supplementary/Figure S8 Correlation between ENC values and G+C content, GC12 and GC3.pdf]
